# A powerful but frequently overlooked role of thermodynamics in environmental microbiology: inspirations from anammox

**DOI:** 10.1128/aem.01668-24

**Published:** 2025-01-06

**Authors:** Zibin Li, Mingda Zhou, Xiaochuan Ran, Weigang Wang, Han Wang, Tong Wang, Yayi Wang

**Affiliations:** 1 State Key Laboratory of Pollution Control and Resources Reuse, College of Environmental Science and Engineering, Tongji University 150797 , Shanghai, China; 2 Shanghai Institute of Pollution Control and Ecological Security, Shanghai, China; Shanghai Jiao Tong University, Shanghai, China

**Keywords:** thermodynamics, anammox, nitrogen-transforming microorganisms, bioenergetics, niche differentiation

## Abstract

Thermodynamics has long been applied in predicting undiscovered microorganisms or analyzing energy flows in microbial metabolism, as well as evaluating microbial impacts on global element distributions. However, further development and refinement in this interdisciplinary field are still needed. This work endeavors to develop a whole-cycle framework integrating thermodynamics with microbiological studies, focusing on representative nitrogen-transforming microorganisms. Three crucial concepts (reaction favorability, energy balance, and reaction directionality) are discussed in relation to nitrogen-transforming reactions. Specifically, reaction favorability, which sheds lights on understanding the diversity of nitrogen-transforming microorganisms, has also provided guidance for novel bioprocess development. Energy balance, enabling the quantitative comparison of microbial energy efficiency, unravels the competitiveness of nitrogen-transforming microorganisms under substrate-limiting conditions. Reaction directionality, revealing the niche-differentiating patterns of nitrogen-transforming microorganisms, provides a foundation for predicting biogeochemical reactions under various environmental conditions. This review highlights the need for a more comprehensive integration of thermodynamics in environmental microbiology, aiming to comprehensively understand microbial impacts on the global environment from micro to macro scales.

## INTRODUCTION

Thermodynamics, one of the time-honored (well-established with a long history) disciplines in natural science, originates from studies on heat engines and has been expanded to various fields in science and engineering (1). It primarily focuses on energy balance in equilibrium systems as well as the direction and extent of natural processes (2, 3). Since Marjory Stephenson, a distinguished pioneer of chemical microbiology, introduced thermodynamic calculations into bacterial metabolism analysis in 1930 (4), thermodynamics has been widely applied in quantitative studies in microbiology, encompassing various metabolic reactions and geomicrobiological processes (5–9).

Over the past decades, thermodynamics has been applied in studying nitrogen cycle, favoring the discoveries of various novel microorganisms as well as understanding of energy and electron flow in microbial metabolism (6, 10–14). Especially, with the aid of thermodynamics, bacteria responsible for novel nitrogen-transforming bioprocesses, including anaerobic ammonium-oxidizing bacteria (AnAOB), complete ammonia-oxidizing bacteria (CAOB), and ferric ammonia-oxidizing bacteria (feammox bacteria), have been discovered and deeply studied (10, 13, 15, 16). Some of these processes have been used in high-efficiency wastewater treatment such as anaerobic ammonium oxidation (anammox) (17, 18). Moreover, energy conversions within living microorganisms can be elucidated by thermodynamics (9, 19–21), which provides in-depth insights into the microbial metabolic strategies under diverse environments (20, 22–25). Thermodynamics also favors the understanding of the distribution of nitrogen species along the redox gradient (26, 27). These knowledges are basic but vital for elucidating the response of nitrogen transformation to the environmental change (28–30).

Anammox is a typical bioprocess, and the discovery of its functional bacteria (AnAOB) was greatly dependent on thermodynamics (6), which further facilitates comprehensive studies pertaining to the metabolism, enzyme function, and niche discovery of AnAOB (12, 31, 32). However, for most nitrogen-transforming microorganisms, thermodynamics application is prone to be overlooked or not been fully utilized: (i) researchers have mostly failed to integrate thermodynamic analyses, even though such analyses can significantly help interpret the observed phenomena, and (ii) only the Gibbs free energy change has been used to judge the feasibility of specific chemical reactions when using thermodynamics. Actually, thermodynamics can be extended far beyond the Gibbs free energy change of chemical reactions; it can shed light on a wide range of perplexing phenomena associated with microbial energy conversion, biochemical reaction (Section 2, the free-energy efficiency), and biogeochemical cycling (Section 3, the *E*_H_-pH diagram) (5, 9, 22, 24, 33–35).

In this review, we provide a framework for thermodynamic analysis in environmental microbiology based on three key concepts—reaction favorability, energy balance, and reaction directionality. These three concepts enable the whole-cycle analysis of environmental microbiology problems ranging from microorganism discovery, metabolic analysis, and niche prediction. Specifically, reaction favorability aids in predicting and understanding diversities of microorganisms; energy balance facilitates the comparison of energy efficiencies and competitiveness between microorganisms; and reaction directionality helps reveal niche-differentiating patterns and discover the natural habitats of microorganisms. Additionally, we propose novel thermodynamics-based methodologies, such as free-energy efficiency-based microbial competitiveness comparison and *E*_H_-pH diagram-based microbial niche prediction. This review highlights that by the whole-cycle (from microscopic metabolic reactions to macroscopic geomicrobiological events) integrations with environmental microbiology, thermodynamics will guide us toward a territory where theoretical principles and practical applications mutually validate, elevating the novelty and scientific robustness of explorations in environmental microbiology.

## THE THERMODYNAMIC FAVORABILITY OF BIOCHEMICAL REACTIONS—THERMODYNAMICS HELP ILLUSTRATE THE DIVERSITY OF NITROGEN-TRANSFORMING MICROORGANISMS

**Fig 1:**
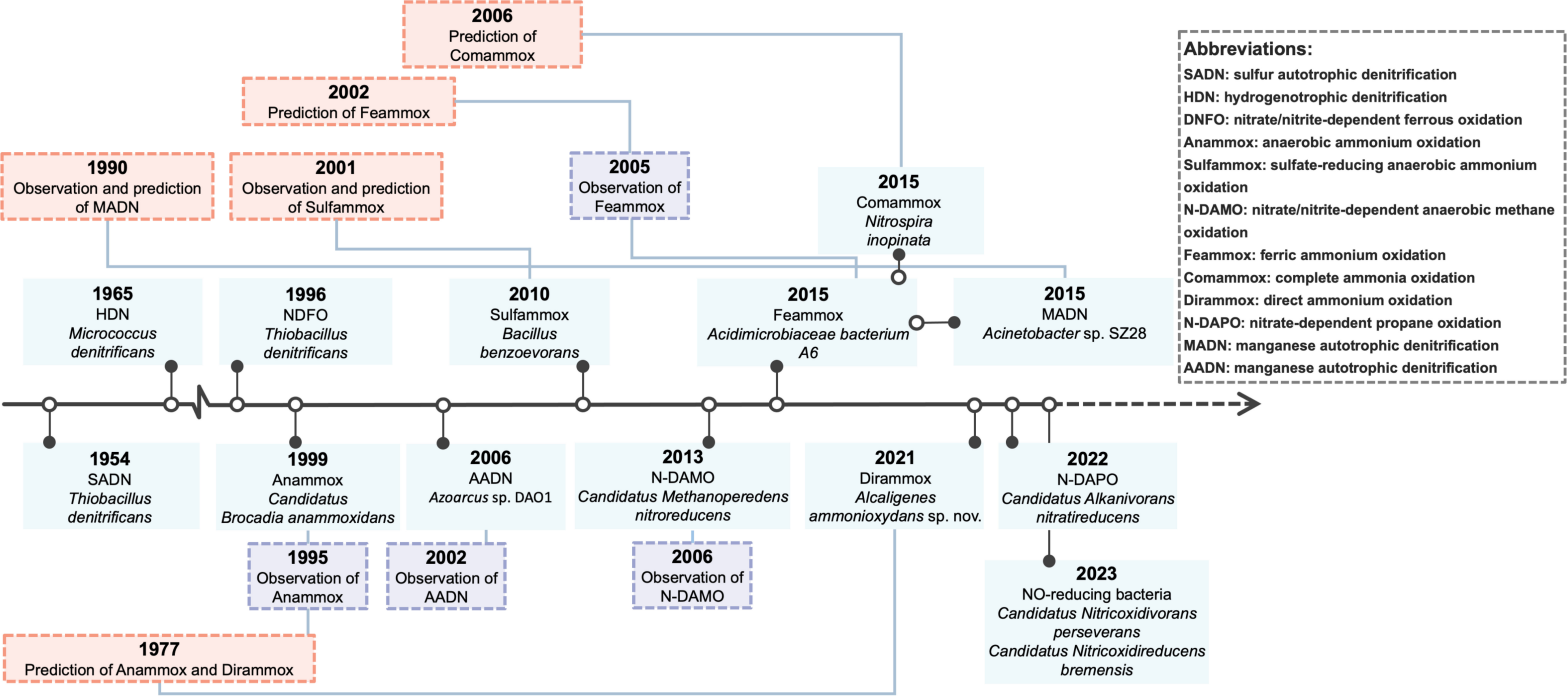
Thermodynamic predictions, observations, and discoveries of typical nitrogen-transforming microorganisms. The timeline shows the major events from 1954 to 2023.

Thermodynamic favorability describes the ability of a reaction to release free energy and proceed toward a more thermodynamically stable state over time. This refers to the system evolving toward equilibrium, where the Gibbs free energy change (Δ*G*) approaches 0 (36). The Gibbs free energy change (∆*G*) of a reaction quantitatively describes its favorability: a more negative ∆*G* indicates greater favorability, while a positive ∆*G* denotes a thermodynamically unfavorable reaction. The electrode potential (*E*(Ox/Red)) is another powerful tool for judging the chemical reaction direction and tendency: a positive electrode potential favors reduction (favorable reduction half-reaction), while a negative electrode potential favors oxidation (favorable oxidation half-reaction) (see the supplemental material) (1). It is important to note that thermodynamic favorability merely indicates the potential for a reaction to occur. Although a reaction may be thermodynamically favorable (Δ*G* < 0), it does not definitely mean a rapid reaction rate, i.e., kinetics can vary significantly depending on environmental conditions and the presence of functional microorganisms. Thermodynamic favorability is not only a fundamental prerequisite for microbial energy conservation but also a catalyst for novel microorganism discoveries (6). Since the 1950s, more than ten diverse nitrogen-transforming microorganisms have been discovered (Fig. 1; Table S1) (15, 16, 37–49). As one of the most ground-breaking nitrogen-cycling pathways, the discovery of anammox has been highly dependent on thermodynamics. The existence of anammox bacteria was predicted by Broda in 1977 before its final discovery in 1999. Broda is an Austrian theoretical chemist; in his paper entitled “two kinds of lithotrophs missing in nature,” he predicted that there might be a certain kind of chemolithotroph that is able to oxidize ammonium coupled with the reduction of nitrite, because the related biochemical reaction is thermodynamically favorable (10, 50):


$$NH_{4}^{+}+NO_{2}^{-}\to N_{2}+2H_{2}O\;\Delta G^{0^{\prime }}=-119\;kJ/mol\;e^{-}.$$

Two decades later, Mulder et al. observed a significant loss of ammonium under anoxic conditions in a denitrifying fluidized reactor treating methanogenic effluent (11); they confirmed the presence of the “missing” lithotroph based on Broda’s prediction and named it “Anammox bacteria.” Other well-known nitrogen-transforming microorganisms such as feammox bacteria, dirammox bacteria, manganese autotrophic denitrifying bacteria, and CAOB, were also predicted to exist in the biosphere by calculating the Gibbs free energy change of their catabolic reactions prior to their discoveries (Table S1). The thermodynamic favorability of various nitrogen-transforming reactions also contributes to the diversity of nitrogen-transforming microorganisms since microorganisms tend to exploit every possible exergonic reaction that can provide energy for their metabolism (10, 13, 32, 51).

**Fig 2:**
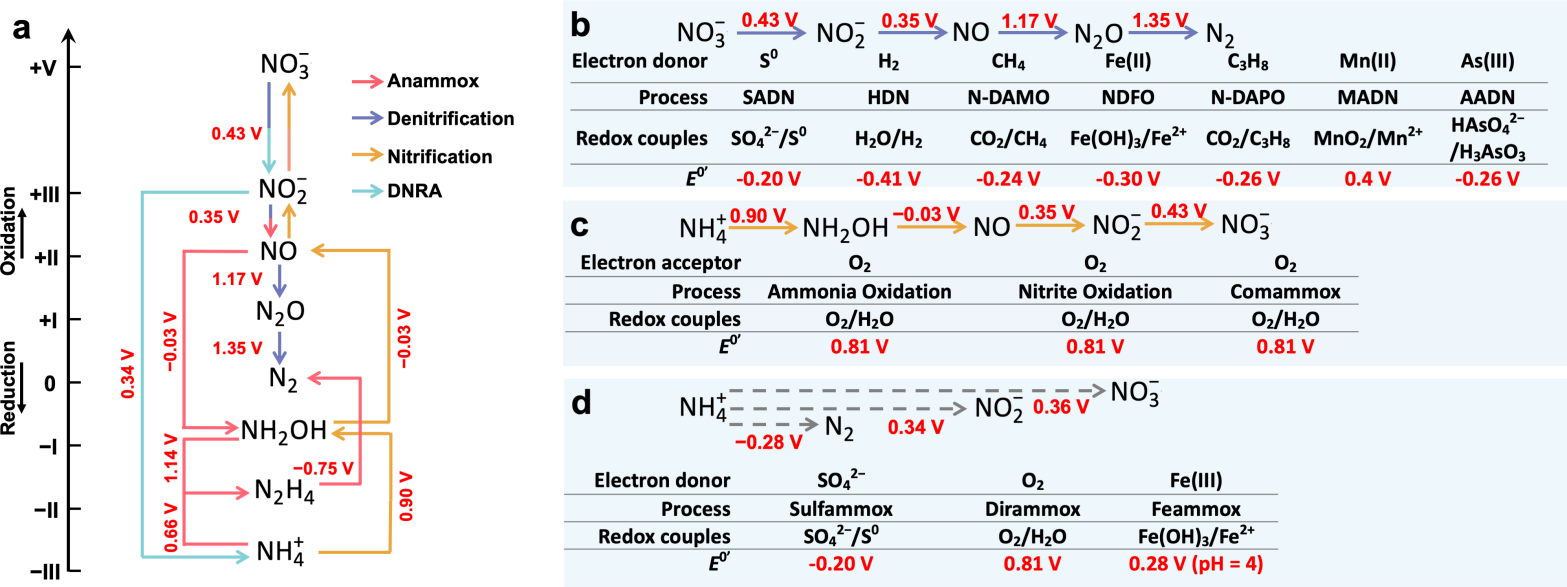
Microbial-mediated interconversions of nitrogen species alongside redox potentials for corresponding half-reactions. (**a**) The nitrogen-transforming network shows the microbially catalyzed half-reactions for nitrogen transformation. (**b**) Existing microbial-mediated denitrification processes with varying electron donors. (**c**) By coupling the downstream favorable reactions (NH_2_OH→NO→NO_2_^−^→NO_3_^−^), ammonia oxidizers are able to overcome the first unfavorable step of ammonia oxidation (NH_4_^+^→NH_2_OH).(**d**) While the intermediates of several processes involved in nitrogen transformations, such as sulfammox, dirammox, and feammox, remain poorly understood, the overall reactions are thermodynamically favorable according to their redox potentials.

Indeed, we are always wondering that how nitrogen-transforming microorganisms have evolved into such a diverse range of species and functions. The answer may lie in the diverse thermodynamically favorable pathways for nitrogen conversion. Generally, the biological nitrogen cycle relies on the conversion of nitrogen species between different oxidation states, driven by various redox reactions. These reactions can be grouped into several thermodynamically feasible pathways via specific nitrogenous intermediates (Fig. 2a) (52). Most nitrogen-transforming processes follow these pathways but differentiate in their specific reaction mechanisms, primarily because microorganisms responsible for these processes have diverse adaptation modes to different niches. For instance, denitrifying bacteria exhibit versatile flexibility in utilizing various organic or inorganic electron donors, such as methane (nitrate-/nitrite-dependent anaerobic methane oxidation, N-DAMO), propane (nitrate-dependent propane oxidation, N-DAPO), reduced sulfur compounds (i.e., elemental sulfur, sulfide, thiosulfate, and sulfite) (sulfur autotrophic denitrification, SADN), ferrous iron (nitrate-dependent ferrous oxidation, NDFO), divalent manganese ion (manganese autotrophic denitrification, MADN), arsenite (arsenic autotrophic denitrification, AADN), and hydrogen (hydrogen autotrophic denitrification, HDN) (37–39, 42, 44, 46, 48). All these biological reactions are thermodynamically favorable under physiological conditions and thus can support the metabolism of denitrifying bacteria under diverse environments (Fig. 2b). Similarly, nitrifying bacteria exhibit different levels of oxidation of nitrogen species, e.g., NH_4_^+^→NH_2_OH→NO→NO_2_^−^ for ammonia-oxidizing bacteria (AOB), NO_2_^−^→NO_3_^−^ for nitrite-oxidizing bacteria (NOB), and NH_4_^+^→NH_2_OH→NO→NO_2_^−^→NO_3_^−^ for CAOB (Fig. 2c) (53). These conversions are all thermodynamically favorable, with molecular oxygen as the electron acceptor. The differences in the metabolic strategy of nitrifying bacteria might further contribute to their adaptative potential to different environments (25, 54). For example, CAOB has a superior capacity to harvest energy from the substrates because it has a longer metabolic pathway (oxidizing ammonia to nitrite and further to nitrate) than that of AOB (oxidizing ammonia to nitrite), ultimately allowing them to be more adaptative to oligotrophic environments, since it can conserve more energy than AOB with limited ammonia supply (55). For some other novel nitrogen-transforming processes, such as ferric ammonium oxidation (feammox), sulfate-reducing ammonium oxidation (sulfammox), and direct ammonium oxidation (dirammox), their mechanisms for energy conservation are still poorly understood (Fig. 2d) (43, 45, 47). Nevertheless, these processes tend to involve known intermediates (e.g., hydroxylamine and nitric oxide) in the nitrogen-transforming network, which adheres to the principle of “Ockham’s razor” (this principle emphasizes the biochemical simplicity and minimization of introduction of novel intermediates) (56).

Actually, almost all combinations of electron donors and electron acceptors can support microbial living if these reactions are coupled to an electron transport chain that drives oxidative phosphorylation to form ATP (51, 57). In the natural environment, nitrogenous species always coexist with other redox-active compounds that provide free energy for certain microorganisms and properly support their growth. There are many nitrogen-transforming reactions that are thermodynamically feasible, but the mediated functional microorganisms remain undiscovered, such as ammonium oxidation to dinitrogen coupled to reduction of arsenate (58) or manganese oxide (59, 60) and ammonium oxidation to nitrate coupled to reduction of manganese oxide (61, 62) or selenate. Therefore, there might be diversity of undiscovered microorganisms existing on the Earth than our imagination, and many of them may play important roles in the nitrogen cycle. We believe that thermodynamics will continue to help uncover more “missing” microorganisms in the future.

## ENERGY CONVERSION BETWEEN CATABOLISM AND ANABOLISM—FREE-ENERGY EFFICIENCIES EXPLAIN THE COMPETITIVENESS OF NITROGEN-TRANSFORMING MICROORGANISMS UNDER SUBSTRATE LIMITATION

Energy balance describes the conservation of energy, which means energy cannot be created or destroyed (36). Energy balance is a valuable tool for analyzing bioenergetic features of microorganisms (i.e., the mechanisms and processes by which these organisms generate, store, and utilize energy) since it can quantitatively describe the energy transformation between biochemical reactions. In the study on anammox biochemistry, thermodynamic analyses have been used to reveal the molecular mechanism of its catabolic reaction as well as the energy flow in its anabolic pathway (56, 63). For instance, thermodynamic analysis challenges the initial hypothesis that anammox bacteria achieve autotrophy through coupling nitrite oxidation with carbon dioxide fixation. Further studies support the thermodynamic analysis that these bacteria derive reducing equivalents from hydrazine dehydrogenation, whereas nitrite oxidation (NO_2_^−^→NO_3_^−^) is actually coupled to nitrite reduction (NO_2_^−^→NO) (see the supplemental material) (12, 31). By quantitatively analyzing the free-energy efficiency (a parameter quantifying the percentage of energy obtained from catabolic reactions that contribute to biomass yields) between catabolism and anabolism of microorganisms, the nature of energy conversions of microorganisms as “tiny machineries” can be clearly elucidated. In this section, free-energy efficiencies of different nitrogen-transforming microorganisms were compared. We found that the free-energy efficiency can provide insights into the competitiveness of microorganisms under substrate-limiting environments (part 2 in this section).

### Thermodynamic analysis elucidates the reasons and mechanisms behind the differences in free-energy efficiency among nitrogen-transforming microorganisms

**Fig 3:**
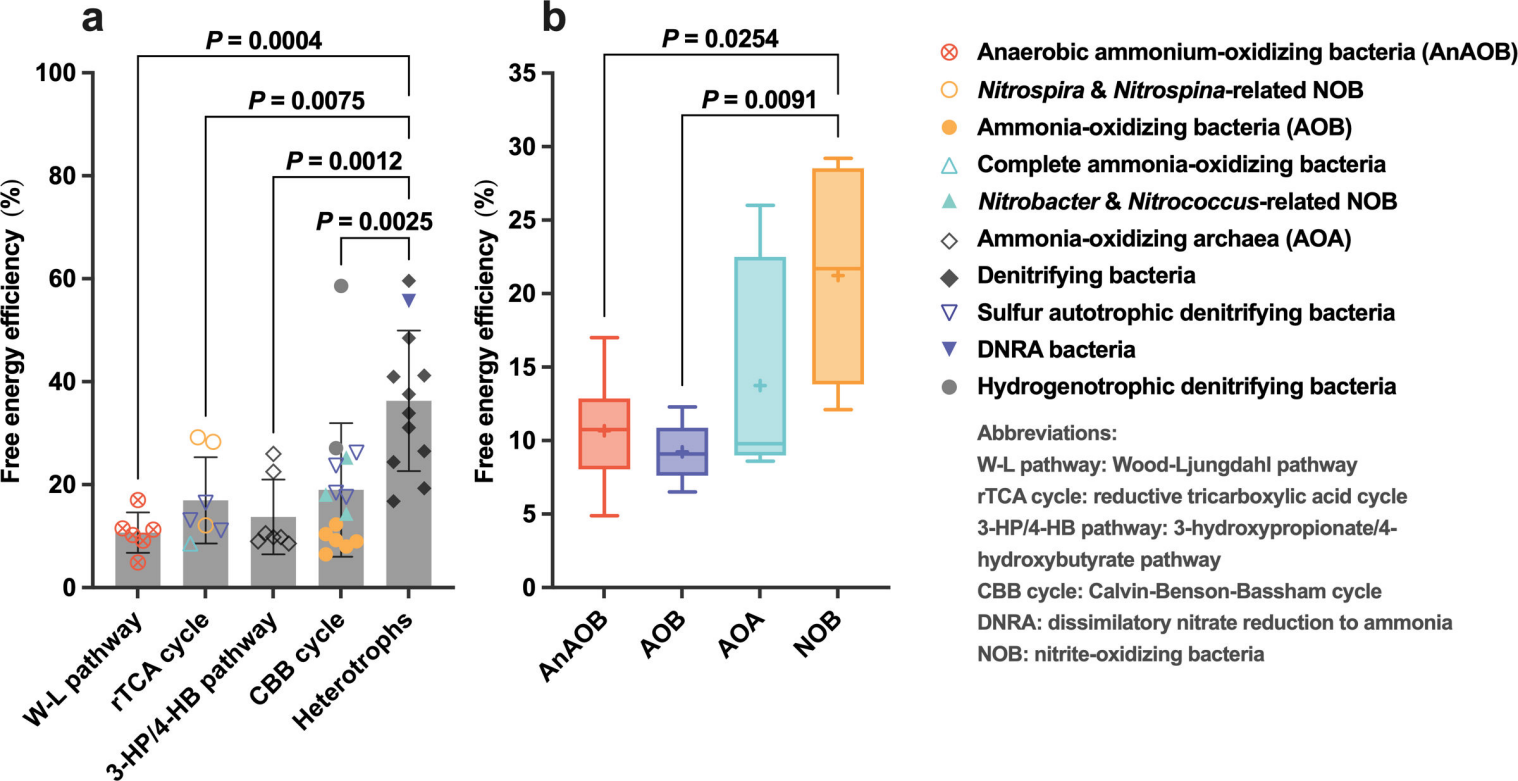
Free-energy efficiencies of different nitrogen-transforming microorganisms. The free-energy efficiencies vary (**a**) among heterotrophs and autotrophs with different autotrophic pathways as well as (**b**) among different autotrophic functional microorganisms. To calculate free-energy efficiencies of diverse groups of nitrogen-transforming microorganisms, a total of 47 experimental yield coefficients (*Y*_obs_) comprising nine metabolic diverse groups of nitrogen-transforming microorganisms were acquired from the previous literature and review articles (Figure 3; Table S3). Pairwise one-way ANOVA tests (Bonferroni method for multiple comparisons) were conducted to assess statistical significance.

For microorganisms, the free-energy efficiency quantifies the percentage of energy obtained from catabolic reactions that contribute to biomass yields (25). In order to compare the free-energy efficiency among different nitrogen-transforming microorganisms, a total of 44 experimental yield coefficients (*Y*_obs_) comprising nine metabolic diverse groups of nitrogen-transforming microorganisms were acquired from the previous literature and review articles (Fig. 3; Table S3). The free-energy efficiencies are calculated based on the following equation:


$$free\;energy\;efficiency\;(\%)=growth\;yield\times \frac{\text{495}}{n\times \Delta G}\times 100.$$

where ∆*G* is the free energy released by catabolic free energy (kJ/mol e^−^) (Tables S4 and S5); *n* represents the number of electrons transferred from the electron donor to the electron acceptor when one mole of electron donor undergoes oxidation; 495 (kJ/mol C) is the average energy required for the reduction of 1 mol of carbon from carbon dioxide to its average oxidation level in cellular constituents (25, 64); growth yields were measured in mol C/mol e^−^ donor.

The free-energy efficiencies differ significantly among different groups of nitrogen-transforming microorganisms (Fig. 3), leading to their different competitiveness under substrate-limiting environments. For example, the free-energy efficiency of heterotrophic nitrogen-transforming microorganisms is significantly higher than that of autotrophic ones (Fig. 3a), which is caused by the different biomass synthesis pattern between autotrophic and heterotrophic microorganisms. For obligate autotrophs, a significant amount of energy is consumed during the process of carbon fixation as reactions converting inorganic carbon to biomass are thermodynamically highly unfavorable (57). However, heterotrophs can directly utilize organic “building blocks” for biomass synthesis without carbon fixation, leading to a higher growth yield in organic-rich environments (65).

Also, the free-energy efficiency differs greatly even among autotrophic nitrogen-transforming microorganisms (Fig. 3). Specifically, the free-energy efficiency of NOB (~20%) is significantly higher than that of AnAOB (~10%, *P* = 0.0225, one-way ANOVA) and AOB (~10%, *P* = 0.0184, one-way ANOVA). This is primarily because NOB dissipate much less energy than AOB and AnAOB, thereby conserving more energy for biomass synthesis. As for AOB, the first step of aerobic ammonia oxidation to hydroxylamine is exergonic but does not couple with proton motive force (PMF) generation (25). Therefore, the free energy released by this reaction (∆*G*^0′^= −153 kJ/mol NH_3_ [see the supplemental material]) is dissipated, leading to a low free-energy efficiency. Regarding AnAOB, energy mainly dissipates during hydrazine dehydrogenation, during which electrons from hydrazine [*E*^0′^(N_2_/N_2_H_4_) = −0.75 V] enter the electron transport chain via diffusive redox partner ferredoxin [*E*^0′^(Fd_ox_/Fd_red_) = −0.42 V] (12, 56, 57). Although this reaction produces 4 H^+^ directly, which contributes to the generation of PMF, the redox potential difference between hydrazine and ferredoxin redox couples is very large (∆*E*^0′^ = 0.33 V); and this potential difference cannot be conserved by generating PMF since hydrazine dehydrogenase is not spatially associated with the anammoxosome membrane (66). Furthermore, electron transfer via diffusive ferredoxin is subject to inherent energy dissipation resulting from diffusion as well as the binding–unbinding reactions with Rieske–heme *b* complexes (67, 68). As a result, a considerable amount of energy is dissipated during electron transfer from hydrazine to the Rieske–heme *b* complexes.

For NOB, by contrast, the oxidation of NO_2_^−^ directly contributes to the generation of PMF, and the electrons released by NO_2_^−^ enter the electron transport chain directly from the membrane-bound nitrite oxidoreductase without diffusive carriers (57). Moreover, NOB possess a very short electron transport chain, with cytochrome *c* acting as the only diffusive electron shuttle, which can minimize energy dissipation during electron transfer (57, 69). Consequently, NOB can obtain more energy from catabolic reactions and yield more biomass when compared with AOB and AnAOB. As shown in Fig. 3b, there is no significant difference in the free-energy efficiency between ammonia-oxidizing archaea (AOA) and NOB (*P* = 0.3601, one-way ANOVA), which might due to the efficient 3-hydroxypropionate/4-hydroxybutyrate (3HP/4HB) pathway of carbon fixation in AOA (25).

### Free-energy efficiency difference helps explain competitiveness under the substrate-limiting environment of nitrogen-transforming microorganisms

We found that nitrogen-transforming microorganisms with high free-energy efficiencies possess a high competitiveness in substrate-limiting environments (25, 70, 71). For example, NOB possess a higher free-energy efficiency (~20%) (25, 72) over AOB (~10%) and AnAOB (~10%), enabling its competitiveness toward substrate-limiting situations even when near the thermodynamic limit (∆*G* ≈ 0 kJ/mol) (73). High competitiveness of NOB in nitrite- and oxygen-limiting conditions has been observed in both natural and engineered ecosystems (74–78). In oceanic oxygen minimum zones, NOB can sustain the consumption of nearly all dissolved oxygen (DO) when DO concentrations fall below ~400 nM, indicating its remarkable competitiveness toward extreme oxygen deficiency (74–76). In domestic wastewater treatment plants, NOB typically occupy a higher abundance compared with AOB (77, 78), despite the fact that nitrite oxidation yields much less energy (∆*G*^0′^ = −74 kJ/mol NO_2_^−^) than ammonia oxidation (∆*G*^0′^ = −275 kJ/mol NH_4_^+^) (in the scenario of domestic wastewater treatment plant, AOB and NOB consume almost same moles of ammonium and nitrite, respectively). A representative example that illustrates the thermodynamic advantage of NOB over AOB in wastewater treatment systems is the mainstream partial nitritation/anammox process, where low DO was maintained to create an oxygen-limiting environment for suppressing the NOB activity. However, achieving a stable partial nitritation based on the consistent suppression of NOB is challenging in mainstream systems due to the thermodynamic advantage of NOB over AOB in oxygen-deficient and ammonium-limiting wastewater (79).

## DIRECTIONALITY OF BIOGEOCHEMICAL REACTIONS—THERMODYNAMICS CREATES THE NICHE-DIFFERENTIATING PATTERNS FOR NITROGEN-TRANSFORMING MICROORGANISMS

Reaction directionality describes the direction that a reversable reaction proceeds under certain condition (e.g., varying pH, temperature, and redox potential) (36). Actually, reaction directionality can help elucidate microbial niche differentiation since it governs the direction of biogeochemical conversions under certain environmental conditions, which in turn regulates natural habitats for nitrogen-transforming microorganisms. The diversity in electron donors and acceptors creates an eco-thermodynamic gradient, which establishes metabolic habitats inhabited by a variety of microbial partners. These habitats exhibit distinct directions of biogeochemical reactions across different regions (26). The eco-thermodynamic gradient, primarily including the electrode potential (*E*_H_) gradient and pH gradient, shapes the modes of biogeochemical reactions and gives rise to the patterns of microbial niche differentiation. In the anammox case, several potential ecological niches of AnAOB were initially predicted based on thermodynamic considerations and macro-ecological field data (32). Currently, finding natural habitats for novel microorganisms mostly depends on labor-intensive sampling and sequencing. Actually, based on the traditional thermodynamic tool—the *E*_H_-pH diagram, one can identify the direction of biogeochemical reactions under different environments and reveal the niche-differentiating pattern for various nitrogen-transforming microorganisms. This may help screen the experimental sites for microbial nitrogen cycling or even help uncover possible ecological niches of undiscovered microorganisms.

### The directions of nitrogen species conversions determine the favorable nitrogen-transforming reactions within different *E*_H_ and pH ranges

**Fig 4:**
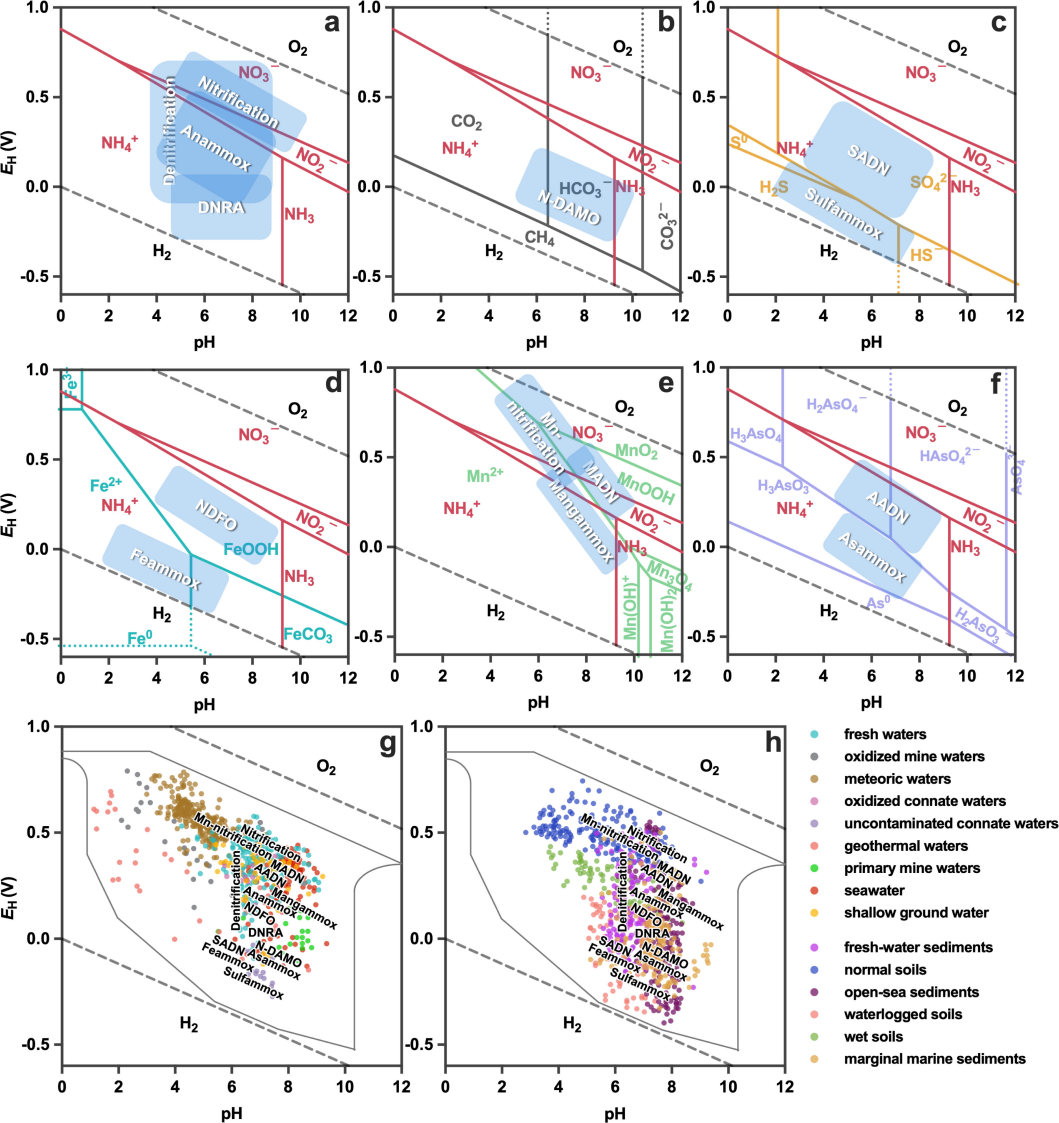
Directionality for nitrogen conversions and potential niche-differentiating patterns of nitrogen-transforming microorganisms influenced by *E*_H_ and pH values. *E*_H_-pH diagrams show the most feasible ranges for (a) typical nitrogen-transforming reactions and (b–f) reactions coupling the nitrogen cycle and other nutrient cycles. Potential niches for different microorganisms are highlighted with blue boxes. The actual niche-differentiation in natural habitats of various nitrogen-transforming microorganisms in (g) natural waters, (h) sediments, and soils are shown. The solid gray line represents the limits of measurements in natural environments proposed by Baas Becking et al. (81). *E*_H_-pH characteristics (points) of natural environments are measured directly from the aquatic and sediment environments, as reported by Baas Becking et al. (81). Abbreviations: Anammox, anaerobic ammonium oxidation; DNRA, dissimilatory nitrate reduction to ammonium; N-DAMO, nitrate-/nitrite-dependent anaerobic methane oxidation; SADN, sulfur autotrophic denitrification; sulfammox, sulfate-reducing anaerobic ammonium oxidation; NDFO, nitrate-/nitrite-dependent ferrous oxidation; feammox, ferric ammonium oxidation; MADN, manganese autotrophic denitrification; AADN, arsenic autotrophic denitrification; Asammox, anaerobic ammonium oxidation coupled with arsenate reduction; Mn-nitrification, Mn(IV)-catalyzed anaerobic nitrification; Mangammox, anaerobic ammonium oxidation linked to Mn(IV) reduction. The *E*_H_-pH diagrams for different elements were constructed using HSC Chemistry 6.00. The physical conditions and total concentrations are set to the typical values in aquatic systems, as shown in Table S6.

The thermodynamic tendency for product formation as well as the availability of substrates governs the thermodynamically feasible *E*_H_-pH ranges for microbial-mediated reactions, which subsequently result in niche-differentiation for microorganisms. Thermodynamically, the *E*_H_-pH diagram describes the predominant chemical species as well as the feasible direction for species conversion under certain *E*_H_ and pH (Fig. 4a through f) (80). Since microorganisms conserve energy from exergonic reactions, they will thrive only within the *E*_H_-pH regions where their catabolic reactions are thermodynamically favorable, which are the product-predominating regions in the *E*_H_-pH diagrams.

The direction of nitrogen species conversions—whether a certain species undergoes oxidation or reduction—depends on the *in situ* physiochemical conditions as well as the coexisting species. As shown in Fig. 4a, nitrification locates slightly above the NH_4_^+^-NO_2_^−^ line where the products (NO_2_^−^ and NO_3_^−^) are thermodynamically stable. Within this range, the substrate (NH_4_^+^) is thermodynamically active, enabling ammonia oxidation by molecular oxygen. The denitrification process occurs around the NH_4_^+^-NO_2_^−^ line and has a wide distribution across the *E*_H_-pH diagram (82–85). This is because dinitrogen gas—the product of denitrification—is considered a redox-inert species, which can be produced under almost any physiological *E*_H_-pH circumstances (see the supplemental material) (80). The anammox process dominates in the range below the NH_4_^+^-NO_2_^−^ line where both NO_2_^−^ and NH_4_^+^ are available (86–90). Dissimilatory nitrite reduction to ammonium (DNRA) mainly dominates far below the NH_4_^+^-NO_2_^−^ boundary where NH_4_^+^ is the predominant nitrogen species (85, 91). This is because the DNRA process has a thermodynamic advantage over heterotrophic denitrification in this nitrate-limiting and organic-rich environment (82).

In the natural environment, the nitrogen cycle is always coupled with other elemental cycles by the biogeochemical reactions between nitrogen and other elements (carbon, sulfur, iron, manganese, and arsenic) (52). As for these interactive biogeochemical reactions, the feasible *E*_H_-pH ranges should consider stabilities of all involved substrates and products as well as substrate coexistence (Fig. 4b through f). For example, the ecological niches for feammox and sulfammox locate primarily in the regions where reduction products (S(−II)/S^0^, Fe(II)) dominate (92–94). This region possess abundant NH_4_^+^, and S(−II)/S^0^ and Fe(II) are thermodynamically stable, favoring ammonium oxidation by S(VI) and Fe(III). On the other hand, niches for denitrifying microorganisms carrying out N-DAMO, SADN, NDFO, manganese autotrophic denitrification (MADN), and arsenic autotrophic denitrification (AADN) locate between the dominant regions of electron donors (CH_4_, S(−II)/S^0^, Fe(II), Mn(II), and As(III)) and NO_3_^−^/NO_2_^−^ (95–98). In these regions, electron donors are thermodynamically unstable (outside their predominant *E*_H_-pH ranges), enabling denitrification by various microorganisms.

### The natural habitats and niche-differentiating patterns revealed by thermodynamics

Actually, determining the favorable *E*_H_-pH ranges is crucial for finding the natural habitats of diverse nitrogen-transforming microorganisms. Specifically, the oxygen divergence among microbial habitats causes the difference in *E*_H_ values, which subsequently determines the direction of nitrogen conversions and microbial niches (28). For example, the surface of freshwaters, seawaters, and soils are usually characterized by high *E*_H_ values (Fig. 4g and h; Table S7) due to the active oxygen transfer from the atmosphere. These aerobic habitats usually receive ammonia fluxes from agricultural runoff, thus enabling the growth of ammonia oxidizers. In contrast, the bottom of aquatic systems such as sediments typically possess low *E*_H_ values (Fig. 4g and h; Table S7), where minimal molecular oxygen or nitrate/nitrite accumulation is observed. These anaerobic habitats mainly possess the reduced form of nitrogen (NH_4_^+^) and commonly receive the downward fluxes of the oxidized form of sulfur (S(VI)), manganese (Mn(IV)), arsenate (As(V)), and iron (Fe(III)), enabling the growth of sulfammox, anaerobic ammonium oxidation linked to Mn(IV) reduction (Mangammox), Mn(IV)-catalyzed anaerobic nitrification (Mn-nitrification), anaerobic ammonium oxidation coupled with arsenate reduction (Asammox), and feammox bacteria that carry out ammonia oxidation with non-oxygen electron acceptors (58, 61, 62). The transitional environments locate between the aerobic and anaerobic environments (e.g., ocean oxygen-minimum zone and sediment–water interface). These habitats receive downward nitrate/nitrate fluxes and upward fluxes of the reduced form of nitrogen (NH_4_^+^), carbon (CH_4_), sulfur (S(0) or S(−II)), iron (Fe(II)), manganese (Mn(II)), and arsenic (As(III)), which will trigger nitrate/nitrite reduction by diverse electron donors, typically including anammox, N-DAMO, SADN, NDFO, MADN, and AADN (Fig. 4g and h; Table S7) (42, 99).

While the *E*_H_-pH diagram provides an overview of potential niches based on measured *E*_H_ and pH values in aquatic environments, it is important to note that *E*_H_ measurements in natural systems using electrochemical sensors cannot be so precise, which mainly stems from three factors: (i) redox couples in natural waters are far from equilibrium, and in this case, the electrode will show mixed potential instead of an equilibrium potential when various redox couples coexist; (ii) probes for redox potential measurement only respond to the redox processes that quickly and reversibly occur at the electrode surface, potentially overlooking the redox couples that are not electroactive; (iii) the functional groups on the solid–liquid interface of small particles in natural waters are easily overlooked, as sensors typically measure only species in the liquid phase (100, 101). Consequently, the *E*_H_-pH diagrams should be interpreted as illustrative of potential niche differentiation rather than precise representations of real-world conditions. Nevertheless, we anticipate that future advancements in measurement techniques will enhance the utility of *E*_H_-pH diagrams in microbial niche prediction.

## DISCUSSION

The thermodynamic concepts (e.g., reaction favorability, energy balance, and reaction directionality) and methodologies (e.g., free-energy efficiency-based microbial competitiveness comparison and *E*_H_-pH diagram-based microbial niche prediction) in this review will help future studies on enriching novel microorganisms and elucidating microbial bioenergetics as well as microbial distribution in diverse natural and engineered systems. By leveraging the power of thermodynamics, researchers can scientifically explain experimental results or even predict undiscovered microbial reactions.

### Developing a thermodynamic mindset in whole-cycle microbiological research

The “thermodynamic mindset” is a conceptual approach where principles from thermodynamics are systematically applied to understand and analyze microbiological processes at different scales. Albert Einstein once said, “Thermodynamics is the only physical theory of universal content, which I am convinced, that within the framework of applicability of its basic concepts will never be overthrown.” This statement implies the absolute correctness of thermodynamic theories within the applicable framework. On the macroscopic scale (Section 3—reaction directionality), biogeochemical cycles encompass various microbial processes that are regulated by thermodynamics. Employing thermodynamics into macroscopic analysis yields valuable insights into the global-scale element distribution (e.g., nitrogen, carbon, and phosphorus) and greenhouse gas production (102). On the microscopic scale, microbial processes that occur in natural or engineered systems are propelled by microbial metabolisms, which predominantly involve substance conversions, energy transformations, and electron transfers within and between individual microorganisms. Incorporating thermodynamics into microscopic analyses facilitates clarification of the energy and electron flows within or between microbial cells (Sections 1 and 2—reaction favorability and energy balance). Furthermore, the thermodynamic mindset can also provide guidance for microbiological experimental design and improve the reliability of the results. Pre-experimental thermodynamic analysis allows predictions of possible outcomes and providing constructive suggestions for subsequent experiments (e.g., microbial niche prediction of microorganism in Section 3). Post-experimental thermodynamic analysis serves to provide robust evidence for microbial phenomena and underlying mechanisms (e.g., energy dissipation analysis in Section 2).

### Relationship between thermodynamic and kinetic considerations in environmental microbiology

Thermodynamics is not the sole factor governing microbial behavior, and kinetics also plays a crucial role in regulation of microbial metabolism, competition, and niche differentiation. A thermodynamically favorable reaction may not sustain microbial growth unless it occurs at a sufficient high rate. For instance, Broda (103) discussed in his book that the reaction involving dinitrogen, dioxygen, and water could be thermodynamically feasible in the biosphere:


$$0.5N_{2}+1.25O_{2}+0.5H_{2}O\to HNO_{3}\;\Delta G^{0^{\prime }}=-6.5\;kJ/mol\;e^{-}.$$

Though this reaction is thermodynamically favorable, there is currently no known enzyme capable of catalyzing it in the biosphere. Moreover, a novel microorganism that catalyzes this reaction could potentially acidify oceans and rivers (6).

Regarding competitiveness and niche differentiation among microorganisms, thermodynamic analysis is only applicable when comparing microorganisms that perform different catabolic reactions (e.g., between anammox bacteria and nitrite oxidizers). However, when comparing different subgroups carrying out the same catabolic reaction, kinetic parameters (e.g., maximum specific growth rate and half-saturation constant) must be considered to obtain more precise and detailed results. For example, anammox bacteria in different genera with varying substrate affinities thrive in different environments. When in the high-nitrite concentration scenario (e.g,. >2 mg NO_2_^−^-N/L), *Candidatus* Brocadia sinica can outcompete most anammox bacteria due to its higher maximum specific growth rate and greater half-saturation constant (104).

In summary, thermodynamics is better suited for determining the feasibility of reactions under specific conditions (“yes or no” issues), while kinetics provides detailed information on reaction rates (“fast or slow” issues) (100). A logical approach in environmental microbiology involves conducting thermodynamic analysis initially to assess feasibility, followed by kinetic analysis to obtain detailed results.

### Advancing thermodynamic models for micro-to-macro microbiological analysis

The integration of quantitative thermodynamic models with either molecular-level or global-scale studies is another promising aspect for bioenergetics and biogeochemistry studies in environmental microbiology. This integration offers a holistic view of material conversions, energy conservations, and entropy productions of biochemical and biogeochemical procedures, helping unravel the molecular mechanisms and global impact of microbial processes (1, 5, 19, 105, 106). For example, thermodynamics models can help predict the microbial growth yield (22), identify the relationship between growth rates and yields of microorganisms (19), as well as offer worldwide quantitative descriptions of microbial activity (5). Since thermodynamics has undergone systematic development, allowing its application to non-equilibrium open systems (36, 107), future models on bioenergetics hold promising opportunities to surpass the limitations of highly simplified “black box” models (50). Moreover, the advanced algorithms and ever-increasing computational capabilities assist in simulating microbial enzyme systems and molecular-level microbial interactions, allowing the prediction of microbial metabolism under diverse environmental scenarios. These advancements also offer a versatile approach to solving global-scale problems, spanning from the effect of microbial conversions on the global element speciation to the complex dynamics of ecosystems and biogeochemical cycles (108).

In the current era driven by big data and machine learning, the future of computational microbiology holds promising opportunities to integrate thermodynamics-based models with global-scale data (e.g., multi-sensor remote sensing data and multiomic sequence information), which will provide valuable knowledge about the global-scale microbial behavior. We believe that an advanced thermodynamic model equipped with comprehensive multidimensional data will provide detailed descriptions of diverse microbial activities and their impact on the global elemental cycle (5, 27, 109, 110).

In conclusion, the path toward achieving a whole-cycle integration of thermodynamics in environmental microbiology is extremely attractive. The exploration of thermodynamic models presents an exciting opportunity for us to unravel the intricate molecular mechanisms that drive microbial behavior across diverse environments. The insights obtained through these efforts will significantly influence the trajectory of environmental microbiology, propelling toward the realization of comprehensive global-scale models that account for the impact of microbes on the global climate change. As researchers delve further into this field, we expect a significant impact on our understanding of the environmental effects of microbial activities, contributing to a more holistic perspective on the global elemental cycles.

## SUPPLEMENTAL MATERIAL

Supplemental material aem.01668-24-s0001.docx Additional details on thermodynamic calculations, analyses, and methods, including tables of the original data.

ASM does not own the copyrights to Supplemental Material that may be linked to, or accessed through, an article. The authors have granted ASM a non-exclusive, world-wide license to publish the Supplemental Material files. Please contact the corresponding author directly for reuse.
