# Supplementary material for: A powerful but frequently overlooked role of thermodynamics in environmental microbiology: inspirations from anammox
Source: Appl Environ Microbiol. 2025 Jan 6;91(2):e01668-24. doi: 10.1128/aem.01668-24 (PMC11837502; doi:10.1128/aem.01668-24)
Supplement: Supplemental material — Additional details on thermodynamic calculations, analyses, and methods, including tables of the original data. [file aem.01668-24-s0001.docx]

Supplemental Material for AEM Minireview

**A powerful but frequently overlooked role of thermodynamics in environmental microbiology: Inspirations from anammox**

Zibin Li,^1,2^ Mingda Zhou,^1,2^ Xiaochuan Ran,^1,2^ Weigang Wang,^1,2^ Han Wang,^1,2*^ Tong Wang,^1,2^ Yayi Wang^1,2*^

^1^State Key Laboratory of Pollution Control and Resources Reuse, College of Environmental Science and Engineering, Tongji University, Siping Road, Shanghai 200092, P. R. China

^2^Shanghai Institute of Pollution Control and Ecological Security, Siping Road, Shanghai 200092, P. R. China

*Corresponding author. Tel: +21 65984275; Fax: +21 65984275 E-mail: wyywater@126.com; yayi.wang@tongji.edu.cn.

**The following are included as supporting information for this paper:**

Number of pages: 35

Number of Tables: 6

Number of Figures: 1

**Supplementary discussions**

**Thermodynamic rules for** **judging the spontaneity** **of a reaction**

*The reaction Gibbs free energy*

When considering chemical reactions under certain condition, the reaction free energy, $\text{∆}\text{G}$, is always adopted to judge the direction of reactions:

$\text{∆}\text{G}$ < 0 for a spontaneous process

$\text{∆}\text{G}$ = 0 for a reversible process

$\text{∆}\text{G}$ > 0 for an impossible process

At fixed *T* and *p*, $\text{∆}\text{G}$can be calculated using the following equation:

$$\text{∆}\text{G}\text{ = }\text{∆}\text{G}^{\text{0}}\text{ + }\text{RT}\text{ln}\text{Q}\text{ = }\text{∆}\text{G}^{\text{0}}\text{ + }\text{RT}\text{ln}\prod_{\text{b}} {\text{a}_{\text{b}}}^{\text{ν}_{\text{b}}}$$

Where $\text{∆}\text{G}^{\text{0}}$ is the standard reaction Gibbs free energy; *R* is the universal gas constant; *Q* is the reaction quotient; *a*_b_ is the activity of species b, *ν*_b_ is the corresponding stoichiometric number of species b.

*The electrode potential*

The electrode potential is another basilic tool for judging the chemical reaction direction and tendency. Considering the chemical reaction between two redox couples, Ox_L_/Red_L_ and Ox_R_/Red_R_:

$$\text{Red}_{\text{L}}\text{ }\text{+}\text{ }\text{Ox}_{\text{R}} \underset{\to}{\text{yields}}{\text{ }\text{Ox}}_{\text{L}}\text{ }\text{+}\text{ }\text{Red}_{\text{R}}$$

The electrochemical cell can be written as:

$$\text{(}\text{-}\text{)}\text{ Ox}_{\text{L}}\text{/}\text{Red}_{\text{L}}\text{ ∥ }\text{Ox}_{\text{R}}\text{/}\text{Red}_{\text{R}}\text{ (+)}$$

Then the standard cell potential is given by:

$$\text{∆}\text{E}^{\text{0}}\text{ = }\text{E}_{\text{R}}^{\text{0}}\left( \text{Ox}_{\text{R}}\text{/}\text{Red}_{\text{R}} \right) \text{- }\text{E}_{\text{L}}^{\text{0}}\text{(}\text{Ox}_{\text{L}}\text{/}\text{Red}_{\text{L}}\text{)}$$

At fixed *T* and *p*, $\text{∆}\text{E}$ can be calculated by Nernst equation:

$$\text{∆}\text{E}\text{ }\text{=}\text{ }\text{∆}\text{E}^{\text{0}} \text{-}\text{ }\frac{\text{RT}}{\text{nF}}\text{ln}\text{Q}$$

Where *F* is Faraday’s constant; *n* is the stoichiometric coefficient of the electrons in the half-reaction into which the cell reaction can be divided.

Like the reaction free energy, $\text{∆}\text{E}$ is widely used to determine the reaction direction:

$\text{∆}\text{E}$ > 0 for a spontaneous process

$\text{∆}\text{E}$ = 0 for a reversible process

$\text{∆}\text{E}$ < 0 for an impossible process

Electrode potentials are more convenient for comparing the thermodynamic tendency between different electron acceptors towards one certain electron donor and vice versa.

*The relation between* $\text{∆}\text{G}$ *and* $\text{∆}\text{E}$

By definition, the reaction Gibbs free energy is equal to the maximum non-expansion work the reaction can perform at constant pressure and temperature. When considering electrical work only, the relation between $\text{∆}\text{G}$ and $\text{∆}\text{E}$ can be given by:

$$\text{∆}\text{G}\text{ }\text{=}\text{ }\text{-}\text{nF}\text{∆}\text{E}$$

This equation builds a bridge between $\text{∆}\text{G}$ and $\text{∆}\text{E}$, showing the equivalence of the two when discussing the direction of reactions. When discussing chemical reaction in natural systems or within cells, $\text{∆}\text{G}^{\text{0'}}\text{ }$or $\text{∆}\text{E}^{\text{0}\text{'}}$ are usually used. The superscript “$\text{0'}$” represent $\text{∆}\text{G}$ or $\text{∆}\text{E}$ calculated at pH = 7 as well as concentrations (more accurately, activity) of all other species equals to unity.

**Thermodynamic feasibility of coupling nitrite oxidation with nitrite reduction in the anammox reaction**

During the anammox reaction, the oxidation of nitrite ($\text{NO}_{\text{2}}^{\text{-}}$→$\text{NO}_{\text{3}}^{\text{-}}$) and reduction of nitrite ($\text{NO}_{\text{2}}^{\text{-}}$→NO) is coupled, in other words, nitrite goes through dismutation to produce nitric oxide and nitrate, as elaborated by the following equation:

|  | $\text{3N}\text{O}_{\text{2}}^{\text{-}}\text{ + }\text{2H}^{\text{+}}\text{ → 2NO + }\text{NO}_{\text{3}}^{\text{-}}\text{ + }\text{H}_{\text{2}}\text{O }\text{∆}\text{G}^{\text{0'}}\text{ = 8.08 kJ/mol }\text{e}^{\text{-}}$ |  |
| --- | --- | --- |

The reaction is thermodynamically unfavorable ($\text{∆}\text{G}^{\text{0'}}$ > 0) under physiological standard conditions (pH = 7, *T* = 298.15 K, unit activities for each species), however, the pH value in the anammoxosome—where the reaction actually take place—is ~6.3(1), which favors the forward reaction ($\text{∆}\text{G}$ = 4.09 kJ/mol e^−^). Besides, the subsequent reaction—nitric oxide reduction to hydroxylamine catalyzed by hydrazine synthase—is thermodynamically favorable. By coupling a thermodynamically slightly unfavorable reaction (nitrite dismutation) with a thermodynamically favorable reaction (nitrite reduction to hydroxylamine), the overall reaction can be thermodynamically favorable(2). In other words, the highly effective cooperation within the tightly coupled multi-component enzyme system can reduce the reaction quotient, further promote this reaction(3).

$\text{Δ}_{\text{r}}\text{G}_{\text{m}}^{\text{0'}}$ **for** **ammonia oxidation to hydroxylamine**

Since ammonia monooxygenase catalyzes the incorporation of only one of the two oxygen atoms of O_2_ into ammonia with the second atom of O_2_ being reduced to H_2_O, ubiquinone (UQH_2_) is required as an additional electron donor in ammonia oxidation. The actual reaction for ammonia oxidation to hydroxylamine is shown below:

$$\text{NH}_{\text{3}}\text{ + }\text{O}_{\text{2}} \text{+ }\text{UQH}_{\text{2}}\text{ → }\text{NH}_{\text{2}}\text{OH}\text{ + }\text{UQ +}\text{ }\text{H}_{\text{2}}\text{O ∆}\text{G}^{\text{0'}}\text{ = -}\text{38.25 kJ/mo}\text{l}\text{ }\text{e}^{\text{-}}$$

**Construction and discussions of the** ***E*_H_-pH diagrams**

*Software and physical conditions*

The *E*_H_-pH diagrams for different elements were constructed with the help of HSC Chemistry 6.00. The physical conditions and total concentrations are set to the typical values in aquatic systems, as shown in Table S6.

*Reasons for neglecting several nitrogen species in the E_H_-pH diagrams*

It has to be pointed out that dinitrogen gas is not considered a redox-active nitrogen species in the *E*_H_-pH diagrams, the reason for which is discussed below. As elaborated by the lg*C*-*E*_H_ and *E*_H_-pH diagram considering all nitrogen species in the biosphere (Figure S1), dinitrogen gas is the predominant species in a broad *E*_H_-pH range, making it thermodynamically unfavorable for other nitrogen species to accumulate in many natural habitats, which seems to contradict the facts. Actually, dinitrogen gas is biologically much less reactive than fixed nitrogen (e.g., ammonium, nitrite, and nitrate), thus neglecting dinitrogen will not affect the equilibrium between other nitrogen species. As a result, the system excluding dinitrogen can be considered a “pseudo-equilibrium” system, which does not contradict the preconditions for constructing an *E*_H_-pH diagram(4).

There are several other nitrogen species that are not included in the *E*_H_-pH diagram, such as hydrazine, hydroxylamine, nitrous oxide, and nitric oxide. The reason for neglecting these species is that they are highly reactive and short-lived, rendering much lower concentrations compared with other fixed nitrogen in natural environments. In addition, some of these species are gases, which makes them easy to escape from aquatic systems. Thus, neglecting these species will also not influence the equilibrium between ammonium, nitrite, and nitrate.

*Advantages of the* *E_H_-pH diagram*

There are several advantages of the *E*_H_-pH diagram. Firstly, the *E*_H_-pH diagram can provide an overview of the predominating species within different *E*_H_-pH ranges. Using this method, one can easily determine the most stable species in a given environment with known *E*_H_-pH characteristics. In addition, the *E*_H_-pH diagram can greatly aid attempts to understand the probable redox patterns, even when the reactions (such as microbial-mediated reactions in aquatic systems) are away from equilibrium(5).

**Statistical analyses**

The statistical significance of differences between different groups of microorganisms for free energy efficiency was assessed using pairwise one-way ANOVA tests with correction for multiple comparisons using the Bonferroni method. The Brown–Forsythe test was used to assess variance in different sample groups for each one-way ANOVA test.

Table S1. Gibbs Free Energy Change and Discovery Processes for Typical Nitrogen-Transforming Processes

| Processes | Reactions | $\text{∆}\text{G}^{\text{0'}}$(kJ/mol e^−^) | Predicted  years | Observed years | Identified years |
| --- | --- | --- | --- | --- | --- |
| SADN | 1.2NO_3_^−^ + S^0^ + 0.4H_2_O → SO_4_^2−^ + 0.6N_2_ + 0.8H^+^ | −91.3 | — | — | 1954(6) |
| HDN | 2NO_3_^−^ + 5H_2_ + 2H^+^ → N_2_ + 6H_2_O | −112.0 | — | — | 1965(7) |
| NDFO | 2NO_3_^−^ + 10Fe^2+^ + 24H_2_O → 10Fe(OH)_3_ + N_2_ + 18H^+^ | −96.2 | — | 1996(8) | 1996(8) |
| Anammox | NH_4_^+^ + NO_2_^–^ → N_2_ + 2H_2_O | −119.0 | 1977(9) | 1995(10) | 1999(11, 12) |
| AADN | 5H_3_AsO_3_ + 2NO_3_^−^ → 5HAsO_4_^2−^ + N_2_ + 8H^+^ + H_2_O | −91.4 |  | 2002(13) | 2006(14) |
| Sulfammox | 2NH_4_^+^ + SO_4_^2−^ → S^0^ + N_2_ + 4H_2_O | −8.0 | 2001(15) | 2001(15) | 2010(16) |
| N-DAMO | 4NO_3_^−^ + CH_4_ → CO_2_ + 4NO_2_^−^ + 2H_2_O | −62.9 | — | 2013(17) | 2013(18) |
| Comammox | NH_4_^+^ + 2O_2_ → NO_3_^−^ + H_2_O + 2H^+^ | −43.6 | 2006(19) | — | 2015(20, 21) |
| Feammox | NH_4_^+^ + 6FeOOH + 10H^+^ → 6Fe^2+^ + 10H_2_O + NO_2_^−^ | −5.2 | 2002(22) | 2005(23) | 2015(24) |
| MADN | 5Mn^2+^ + 2NO_3_^−^ + 4H_2_O → 5MnO_2_ + N_2_ + 8H^+^ | −33.4 | 1990(25) | 1990(25) | 2015(26) |
| Dirammox | 4NH_4_^+^ + 3O_2_ → 6H_2_O + 2N_2_ + 4H^+^ | −105.0 | 1977(9) | — | 2021(27) |
| N-DAPO | 10NO_3_^−^ + C_3_H_8_ → 10NO_2_^−^ + 3CO_2_ + 4H_2_O | −67.4 | — | — | 2022(28) |
| NO-reducing bacteria | 2NO + 2HCOO^−^ + 2H^+^ → N_2_ + 2CO_2_ + 2H_2_O | −91.8 | — | — | 2023(29) |

Abbreviations: SADN, sulfur autotrophic denitrification; HDN, hydrogenotrophic denitrification; DNFO, nitrate-dependent ferrous oxidation; Anammox, anaerobic ammonium oxidation; Sulfammox, sulfate-reducing anaerobic ammonium oxidation; N-DAMO, nitrate/nitrite-dependent anaerobic methane oxidation; Feammox, ferric ammonium oxidation; Comammox, complete ammonia oxidation; Dirammox, direct ammonia oxidation; N-DAPO, nitrate-dependent propane oxidation; MADN, manganese autotrophic denitrification; AADN, arsenic autotrophic denitrification.

Table S2. Carbon fixation pathways carried out by different groups of nitrogen-transforming microorganisms

| Pathways for carbon dioxide fixation | Groups of nitrogen-transforming microorganisms |
| --- | --- |
| 3-hydroxyproprionate/4-hydroxybutarate pathways | Ammonia-oxidizing archaea(30) |
| Calvin-Bassham-Benson cycle | Ammonia-oxidizing bacteria(31), *Nitrobacter*(32), *Nitrococcus*(32), *Nitrolancea*(32), *Nitrotoga*(32), *Paracoccus denitrificans*(33), *Thiobacillus denitrificans*(34) |
| Wood-Ljungdahl pathway | Anaerobic ammonium-oxidizing bacteria(35) |
| reductive tricarboxylic acid cycle | *Nitrospira*(31), *Nitrospina*(31), *Thiomicrospira denitrifican**s*(1–15)(1–5)(36) |

Table S3. Growth yields and free energy efficiencies of various nitrogen-transforming microorganisms

| Microorganisms | Electron donors (concentrations (mM)) /Oxidation products (concentrations (mM)) | Electron acceptors (concentrations (mM))  /Reduction products (concentrations (mM)) | $\text{∆}\text{G}$  (kJ/mol e^−^) | *Y*_obs_ in literatures | Units | Free energy efficiency (%) | References |
| --- | --- | --- | --- | --- | --- | --- | --- |
| **Denitrifying bacteria** |  |  |  |  |  |  |  |
| *Hyphomicrobium* sp. | Ethanol (10)/HCO_3_^−^ (10) | NO_3_^−^ (10)/N_2_ (0.6) | -104.8 | 24 | g cell/mol e^−^ donor | 37.6 | (37) |
| *Campylobacter sputorum* | Lactate (10)/HCO_3_^−^ (10) | NO_3_^−^ (10)/N_2_ (0.6) | -106.8 | 20.2 | g cell/mol e^−^ donor | 31.1 | (37) |
| *Paracoccus denitrificans* | Acetate (10)/HCO_3_^−^ (10) | NO_3_^−^ (10)/N_2_ (0.6) | -101.6 | 17 | g cell/mol e^−^ donor | 41.2 | (37) |
| *Paracoccus denitrificans* | Formate (10)/HCO_3_^−^ (30) | NO_3_^−^ (10)/N_2_ (0.6) | -112.0 | 3.03 | g cell/mol e^−^ donor | 26.5 | (38) |
| *Paracoccus denitrificans* | Acetate (5)/HCO_3_^−^ (30) | NO_2_^−^ (2)/N_2_ (0.6) | -117.8 | 23.2 | g cell/mol e^−^ donor | 48.5 | (38) |
| *Paracoccus denitrificans* | Formate (20)/HCO_3_^−^ (30) | NO_2_^−^ (2)/N_2_ (0.6) | -129.1 | 3.2 | g cell/mol e^−^ donor | 24.4 | (38) |
| *Pseudomonas stutzeri* | Acetate (2)/HCO_3_^−^ (10) | NO_3_^−^ (10)/N_2_ (0.6) | -101.6 | 16.9 | g cell/mol e^−^ donor | 41.0 | (37) |
| *Pseudomonas stutzeri* | Acetate (5)/HCO_3_^−^ (30) | NO_2_^−^ (2)/N_2_ (0.5) | -117.8 | 28.5 | g cell/mol e^−^ donor | 59.6 | (38) |
| **DNRA bacteria** |  |  |  |  |  |  |  |
| *Wollinella succinogenes* | Formate (10)/HCO_3_^−^ (30) | NO_3_^−^ (5)/NH_4_^+^ (1) | -73.4 | 4.15 | g cell/mol e^−^ donor | 55.7 | (38) |
| **Hydrogenotrophic denitrifying bacteria** |  |  |  |  |  |  |  |
| *Paracoccus* sp. | H_2_ (0.6)/H_2_O | NO_3_^−^ (3)/N_2_ (0.6) | -101.8 | 6.05 | g cell/mol H_2_ | 58.6 | (39) |
| *Paracoccus denitrificans* | H_2_ (0.6)/H_2_O | NO_3_^−^ (3)/N_2_ (0.6) | -101.8 | 2.8 | g cell/mol H_2_ | 27.1 | (38) |
| **Anaerobic ammonium-oxidizing bacteria** |  |  |  |  |  |  |  |
| *Ca. Brocadia sinica* | NH_4_^+^ (2)/N_2_ (0.6) | NO_2_^−^ (0.2)/N_2_ (0.6) | -101.8 | 0.063 | mol C/mol N | 10.2 | (40) |
| *Ca. Brocadia anammoxidans* | NH_4_^+^ (2)/N_2_ (0.6) | NO_2_^−^ (0.2)/N_2_ (0.6) | -101.8 | 0.07 | mol C/mol N | 11.3 | (40) |
| *Ca. Brocadia caroliniensis* | NH_4_^+^ (2)/N_2_ (0.6) | NO_2_^−^ (0.2)/N_2_ (0.6) | -101.8 | 0.105 | mol C/mol N | 17.0 | (40) |
| *Ca. Brocadia* sp. 40 | NH_4_^+^ (2)/N_2_ (0.6) | NO_2_^−^ (0.2)/N_2_ (0.6) | -101.8 | 0.071 | mol C/mol N | 11.5 | (40) |
| *Ca. Jettenia caeni* | NH_4_^+^ (2)/N_2_ (0.6) | NO_2_^−^ (0.2)/N_2_ (0.6) | -101.8 | 0.056 | mol C/mol N | 9.1 | (40) |
| *Ca. Scalindua* sp. | NH_4_^+^ (2)/N_2_ (0.6) | NO_2_^−^ (0.2)/N_2_ (0.6) | -101.8 | 0.03 | mol C/mol N | 4.9 | (40) |
| **Ammonia-oxidizing archaea** |  |  |  |  |  |  |  |
| *Nitrososphaera gargensis* | NH_4_^+^ (1)/NO_2_^−^ (1) | O_2_ (0.15)/H_2_O | -40.4 | 0.205 | g VSS/g N | 26.0 | (41) |
| *Nitrosopumilus piranensis* D3C | NH_4_^+^ (1)/NO_2_^−^ (1) | O_2_ (0.15)/H_2_O | -40.4 | 1.2 | g cell/mol NH_4_^+^ | 9.8 | (42) |
| *Nitrosopumilus adriaticus* NF5 | NH_4_^+^ (1)/NO_2_^−^ (1) | O_2_ (0.15)/H_2_O | -40.4 | 1.2 | g cell/mol NH_4_^+^ | 9.8 | (42) |
| *Nitrosopumilus maritimus* SCM1 | NH_4_^+^ (1)/NO_2_^−^ (1) | O_2_ (0.15)/H_2_O | -40.4 | 1.3 | g cell/mol NH_4_^+^ | 10.6 | (43) |
| *Nitrosopumilus maritimus* HCA1 | NH_4_^+^ (1)/NO_2_^−^ (1) | O_2_ (0.15)/H_2_O | -40.4 | 1.1 | g cell/mol NH_4_^+^ | 9.0 | (43) |
| *Nitrosopumilus maritimus* PS0 | NH_4_^+^ (1)/NO_2_^−^ (1) | O_2_ (0.15)/H_2_O | -40.4 | 1.06 | g cell/mol NH_4_^+^ | 8.6 | (43) |
| *Ca. Nitrosopelagicus brevis* U25 | NH_4_^+^ (1)/NO_2_^−^ (1) | O_2_ (0.15)/H_2_O | -40.4 | 0.11 | mol C/mol NH_4_^+^ | 22.5 | (31) |
| **Complete ammonia-oxidizing bacteria** |  |  |  |  |  |  |  |
| *Nitrospira inopinata* | NH_4_^+^ (0.5)/NO_3_^−^(0.5) | O_2_ (0.15)/H_2_O | -38.2 | 397.4^a^ | μg protein/mol NH_4_^+^ | 8.6 | (44) |
| **Ammonia-oxidizing bacteria** |  |  |  |  |  |  |  |
| *Nitrosomonas europaea* | NH_4_^+^ (0.5)/NO_2_^−^ (0.5) | O_2_ (0.15)/H_2_O | -40.4 | 0.063 | g biomass/g NH_4_^+^ | 8.0 | (45) |
| *Nitrosomonas marina* | NH_4_^+^ (5)/NO_2_^−^ (5) | O_2_ (0.15)/H_2_O | -40.4 | 0.06 | mol C/mol NH_4_^+^ | 12.3 | (46) |
| *Nitrosococcus oceanus* | NH_4_^+^ (5)/NO_2_^−^ (5) | O_2_ (0.15)/H_2_O | -40.4 | 0.051 | mol C/mol NH_4_^+^ | 10.4 | (46) |
| *Nitrosococcus oceani* | NH_4_^+^ (0.5)/NO_2_^−^ (0.5) | O_2_ (0.15)/H_2_O | -40.4 | 0.8 | g cell/mol NH_4_^+^ | 6.5 | (30) |
| *Nitrosomonas* sp. C-15 | NH_4_^+^ (0.5)/NO_2_^−^ (0.5) | O_2_ (0.15)/H_2_O | -40.4 | 0.044 | mol C/mol NH_4_^+^ | 9.0 | (31) |
| *Nitrosomonas marina* C-25 | NH_4_^+^ (0.5)/NO_2_^−^ (0.5) | O_2_ (0.15)/H_2_O | -40.4 | 0.045 | mol C/mol NH_4_^+^ | 9.2 | (31) |
| **Nitrite-oxidizing bacteria** |  |  |  |  |  |  |  |
| *Nitrobacter agilis* | NO_2_^−^ (7.5)/NO_3_^−^ (7.5) | O_2_ (0.15)/H_2_O | -31.6 | 0.58 | g cell/mol NO_2_^−^ | 18.1 | (47) |
| *Nitrobacter* sp. | NO_2_^−^ (1)/NO_3_^−^ (1) | O_2_ (0.15)/H_2_O | -31.6 | 0.812 | g cell/mol NO_2_^−^ | 25.3 | (48) |
| *Nitrospira calida* | NO_2_^−^ (2)/NO_3_^−^ (2) | O_2_ (0.15)/H_2_O | -31.6 | 0.025 | g VSS/g N | 12.1 | (41) |
| *Nitrospina* sp. Nb-3 | NO_2_^−^ (0.5)/NO_3_^−^ (0.5) | O_2_ (0.15)/H_2_O | -31.6 | 0.0373 | mol C/mol NO_2_^−^ | 29.2 | (31) |
| *Nitrospira marina* Nb-295 | NO_2_^−^ (0.5)/NO_3_^−^ (0.5) | O_2_ (0.15)/H_2_O | -31.6 | 0.0361 | mol C/mol NO_2_^−^ | 28.2 | (31) |
| *Nitrococcus mobilis* Nb-231 | NO_2_^−^ (0.5)/NO_3_^−^ (0.5) | O_2_ (0.15)/H_2_O | -31.6 | 0.0184 | mol C/mol NO_2_^−^ | 14.4 | (31) |
| **Sulfur autotrophic denitrifying bacteria** |  |  |  |  |  |  |  |
| *Thiobacillus denitrificans* | S_2_O_3_^2−^ (10)/SO_4_^2−^ (10) | NO_3_^−^ (20)/N_2_ (0.7) | -96.5 | 9.28 | g cell/mol S_2_O_3_^2−^ | 23.7 | (49) |
| *Thiomicrospira* sp. CVO | HS^−^ (0.5)/SO_4_^2−^ (10) | NO_3_^−^ (1)/N_2_ (0.7) | -88.5 | 6.6 | g cell/mol HS^−^ | 18.4 | (50) |
| *Thiobacillus denitrificans* | HS^−^ (0.1)/SO_4_^2−^ (10) | NO_3_^−^ (5)/N_2_ (0.7) | -88.5 | 9.41 | g cell/mol HS^−^ | 26.2 | (49) |
| *Thiomicrospira denitrificans* | S_2_O_3_^2−^ (10)/SO_4_^2−^ (10) | NO_3_^−^ (20)/N_2_ (0.7) | -96.5 | 5.13 | g cell/mol S_2_O_3_^2−^ | 13.1 | (49) |
| *Thiomicrospira denitrificans* | HS^−^ (0.1)/SO_4_^2−^ (10) | NO_3_^−^ (20)/N_2_ (0.7) | -88.5 | 5.93 | g cell/mol HS^−^ | 16.5 | (49) |
| *Thiobacillus denitrificans* | S_2_O_3_^2−^ (10)/SO_4_^2−^ (10) | NO_2_^−^ (1)/N_2_ (0.7) | -114.3 | 8.15 | g cell/mol S_2_O_3_^2−^ | 17.6 | (49) |
| *Thiomicrospira denitrificans* | S_2_O_3_^2−^ (10)/SO_4_^2−^ (10) | NO_2_^−^ (1)/N_2_ (0.7) | -114.3 | 5.17 | g cell/mol S_2_O_3_^2−^ | 11.1 | (49) |

Factors for units conversions: 1.8g dry weight/g protein(51); 0.9g biomass/g dry weight(45); 0.9g VSS/g dry weight(51); 25.7 g biomass/mol C for anaerobic ammonium-oxidizing bacteria(52); 22.8 g biomass/mol C for other bacteria(51).

^a^The yield coefficient of *Nitrospira inopinata* is converted by assuming it has the same protein content as *Nitrosococcus oceani*, that is, *Y*_obs_ (*Nitrospira inopinata*, mol C/mol NH_4_^+^) = *Y*_obs_ (*Nitrospira inopinata*, μg protein/mol NH_4_^+^) × *Y*_obs_ (*Nitrosococcus oceani*, mol C/mol NH_4_^+^)/*Y*_obs_ (*Nitrosococcus oceani*, μg protein/mol NH_4_^+^)

Table S4. *In-situ* Gibbs free energy changes of various nitrogen-transforming reactions for free-energy efficiency calculation

| Reactions | $\text{∆}\text{G}$ (kJ/mol e^−^) |
| --- | --- |
| **Heterotrophic denitrification** |  |
| CH_3_CH_2_OH + 2.4NO_3_^−^ + 0.4H^+^ → 2HCO_3_^−^ + 1.2N_2_(aq) + 2.2H_2_O | −104.8(37) |
| CH_3_CHOHCOO^−^ + 2.4NO_3_^−^ + 0.4H^+^ → 3HCO_3_^−^ + 1.2N_2_(aq) + 1.2H_2_O | −106.8(37) |
| CH_3_COO^−^ + 1.6NO_3_^−^ + 0.6H^+^ → 2HCO_3_^−^ + 0.8N_2_(aq) + 0.8H_2_O | −101.6(37) |
| HCOO^−^ + 0.4NO_3_^−^ + 0.4H^+^ → HCO_3_^−^ + 0.2N_2_(aq) + 0.2H_2_O | −112(37) |
| **Dissimilatory nitrate reduction to ammonium** |  |
| HCOO^-^ + 0.25NO_3_^−^ + 0.25H_2_O + 0.5H^+^ → HCO_3_^−^ + 0.25NH_4_^+^ | −75(37) |
| **Hydrogenotrophic denitrification** |  |
| H_2_(aq) + 0.4NO_3_^−^ + 0.4H^+^ → 0.2N_2_(aq) + 1.2H_2_O | −113(37) |
| **Anaerobic ammonium oxidation** |  |
| NH_4_^+^ + NO_2_^–^ → N_2_(aq) + 2H_2_O | −118 |
| **Ammonia oxidation** |  |
| NH_4_^+^ + 1.5O_2_ → NO_2_^–^ + H_2_O + 2H^+^ | −41.7 |
| **Nitrite oxidation** |  |
| NO_2_^–^ + 0.5O_2_ →NO_3_^−^ | −35 |
| **Complete ammonia oxidation** |  |
| NH_4_^+^ + 2O_2_ → NO_3_^−^ + H_2_O + 2H^+^ | −39.2 |
| **Sulfur autotrophic denitrification** |  |
| HS^−^ + 1.6NO_3_^−^ + 0.6H^+^ → SO_4_^2−^ + 0.8N_2_(aq) + 0.8H_2_O | −90.4 |
| S_2_O_3_^2−^ + 1.6NO_3_^−^ + 0.2H_2_O → 2SO_4_^2−^ + 0.8N_2_(aq) + 0.4H^+^ | −97.6 |
| S_2_O_3_^2−^ + 2.67NO_2_^−^ + 0.67H^+^ → 2SO_4_^2−^ + 1.33N_2_(aq) + 0.33H_2_O | −117.6 |

Table S5. Species and parameters for constructing the *E*_H_-pH diagrams

| Elements | Species | Total concentration (mol/L) | Temperature (K) | Pressure (kPa) |
| --- | --- | --- | --- | --- |
| N | $\text{NH}_{\text{4}}^{\text{+}}$, $\text{NO}_{\text{2}}^{\text{-}}$, $\text{NO}_{\text{3}}^{\text{-}}$ | 1 × 10^−4^ | 298.15 | 100 |
| C | $\text{CO}_{\text{2}}$, $\text{CO}_{\text{3}}^{\text{2-}}$, $\text{HCO}_{\text{3}}^{\text{-}}$, $\text{CH}_{\text{4}}$ | 4 × 10^−3^ | 298.15 | 100 |
| S | $\text{S}^{\text{0}}$, $\text{S}^{\text{2-}}$, $\text{H}\text{S}^{\text{-}}$, $\text{SO}_{\text{2}}$, $\text{S}_{\text{2}}\text{O}_{\text{3}}^{\text{2-}}$, $\text{HSO}_{\text{4}}^{\text{-}}$, $\text{SO}_{\text{3}}^{\text{2-}}$, $\text{SO}_{\text{4}}^{\text{2-}}$, $\text{FeS}_{\text{2}}$ | 2 × 10^−3^ | 298.15 | 100 |
| Fe | $\text{Fe}^{\text{0}}$, FeO, $\text{Fe}^{\text{2+}}$, $\text{FeCO}_{\text{3}}$, $\text{Fe(OH)}_{\text{2}}$, $\text{Fe}^{\text{3+}}$, $\text{Fe(OH)}_{\text{3}}$, $\text{Fe(OH)}^{\text{+}}$, FeOOH, $\text{Fe}_{\text{3}}\text{O}_{\text{4}}$, $\text{Fe(OH)}_{\text{2}}^{\text{+}}$, $\text{Fe(OH)}_{\text{4}}^{\text{-}}$, $\text{FeS}_{\text{2}}$ | 3 × 10^−3^ | 298.15 | 100 |
| Mn | $\text{Mn}^{\text{0}}$, $\text{Mn}^{\text{2+}}$, MnOOH, $\text{Mn}_{\text{3}}\text{O}_{\text{4}}$, $\text{MnCO}_{\text{3}}$, $\text{Mn(OH)}_{\text{2}}$, | 2 × 10^−6^ | 298.15 | 100 |
| As | $\text{As}^{\text{0}}$, $\text{As}_{\text{2}}\text{S}_{\text{3}}$, $\text{As}_{\text{4}}\text{S}_{\text{4}}$, $\text{AsO}_{\text{4}}^{\text{3-}}$, $\text{AsO}_{\text{3}}^{\text{2-}}$ | 2 × 10^−6^ | 298.15 | 100 |

Table S6. Thermodynamics-Predicted Habitats by *E*_H_-pH diagrams and Documented Natural Habitats of Various Nitrogen-Transforming Microorganisms

| processes | Typical reaction(s) | $\text{∆}\text{G}^{\text{0'}}$  (kJ/mol e^−^) | Thermodynamics-predicted habitats | Identified habitats |
| --- | --- | --- | --- | --- |
| Nitrification | NH_4_^+^ + 1.5O_2_ → NO_2_^–^ + H_2_O + 2H^+^  NO_2_^–^ + 0.5O_2_ →NO_3_^−^ | −45.8  −37.0 | Meteoric waters, freshwaters, seawaters, shallow ground waters, normal soils | Oxic zones of oceans, freshwaters, and soils(53, 54) |
| Heterotrophic  denitrification | 8NO_3_^−^ + 5CH_3_COO^−^ + 3H^+^ → 10HCO_3_^−^ + 4N_2_ + 4H_2_O | −99.5(55) | Almost any habitat | wetland soils(56), freshwater sediment(56), aquaculture sediments(57), ocean oxygen-minimum zones(55), estuarine benthic habitats(58) |
| Anammox | NH_4_^+^ + NO_2_^–^ → N_2_ + 2H_2_O | −119.0(3) | Seawaters, freshwaters, wet soils, marine sediments, freshwater sediments, geothermal waters, primary mine waters | ocean oxygen-minimum zones, marine sediments(55), hypersaline sulphidic basins(59), deep-sea hydrothermal vents(60), freshwater sediments(61), hot springs(62), paddy soils(63), and estuarine benthic habitats(58) |
| DNRA | NO_3_^−^ + CH_3_COO^−^ + H^+^ → 2HCO_3_^−^ + NH_4_^+^ | −102.8(55) | Uncontaminated connate waters, primary mine waters, water-logged soils, marine sediments, freshwater sediments | wetland soils(56), freshwater sediment(56), aquaculture sediments(57), ocean oxygen-minimum zones(55), estuarine benthic habitats(58), groundwaters(64) |
| N-DAMO | 4NO_3_^−^ + CH_4_ → CO_2_ + 4NO_2_^−^ + 2H_2_O  3CH_4_ + 8NO_2_^−^ + 8H^+^ → 3CO_2_ + 4N_2_ + 10H_2_O | −62.9(18)  −116(65) | Uncontaminated connate waters, primary mine waters, water-logged soils, marine sediments, freshwater sediments | Marine sediments(66), freshwater sediment(67, 68), wetland solis(69), paddy soils(70) |
| SADN | 1.2NO_3_^−^ + S^0^ + 0.4H_2_O → SO_4_^2−^ + 0.6N_2_ + 0.8H^+^ | −91.3 | Wet soils, marine sediments, freshwater sediments, shallow groundwaters | ocean oxygen-minimum zones(55), freshwater sediments(71), groundwaters(72), wetlands(73) |
| Sulfammox | 2NH_4_^+^ + SO_4_^2−^ → S^0^ + N_2_ + 4H_2_O  8NH_4_^+^ + 3SO_4_^2−^ → 3HS^−^ + 4N_2_ + 12H_2_O + 5H^+^ | −8.0(15)  −8.9 | Water-logged soils, marine sediments, freshwater sediments, uncontaminated connate waters | Marine sediments(74) |
| DNFO | 2NO_3_^−^+ 10Fe^2+^ + 24H_2_O → 10Fe(OH)_3_ + N_2_ + 18H^+^ | −96.2 | Wet soils, marine sediments, freshwater sediments | Freshwater sediments(75), shallow beach sediments(76) |
| Feammox | NH_4_^+^ + 6FeOOH + 10H^+^ → 6Fe^2+^ + 10H_2_O + NO_2_^−^ | −5.15 | Water-logged soils, marine sediments, freshwater sediments, uncontaminated connate waters | Wetland soils(77), tropical forest soils(78), paddy soils(78), marine sediments(74) |
| MADN | 5Mn^2+^ + 2NO_3_^−^ + 4H_2_O → 5MnO_2_ + N_2_ + 8H^+^ | −33.4 | Marine sediments, freshwater sediments, geothermal waters, shallow groundwaters | Marine sediment-water interface(79), agricultural soils(80), paddy soils(80), groundwaters(81) |
| AADN | H_3_AsO_3_ + 2NO_3_^−^ → HAsO_4_^2−^ + 2NO_2_^−^ + 2H^+^ | −33.5 | Shallow groundwaters, wet soils, marine sediments, freshwater sediments | Flooded paddy soils(82), anoxic lake waters(83) |

Abbreviations: SADN, sulfur autotrophic denitrification; HDN, hydrogenotrophic denitrification; DNFO, nitrate-dependent ferrous oxidation; Anammox, anaerobic ammonium oxidation; Sulfammox, sulfate-reducing anaerobic ammonium oxidation; N-DAMO, nitrate/nitrite-dependent anaerobic methane oxidation; Feammox, ferric ammonium oxidation; MADN, manganese autotrophic denitrification; AADN, arsenic autotrophic denitrification.


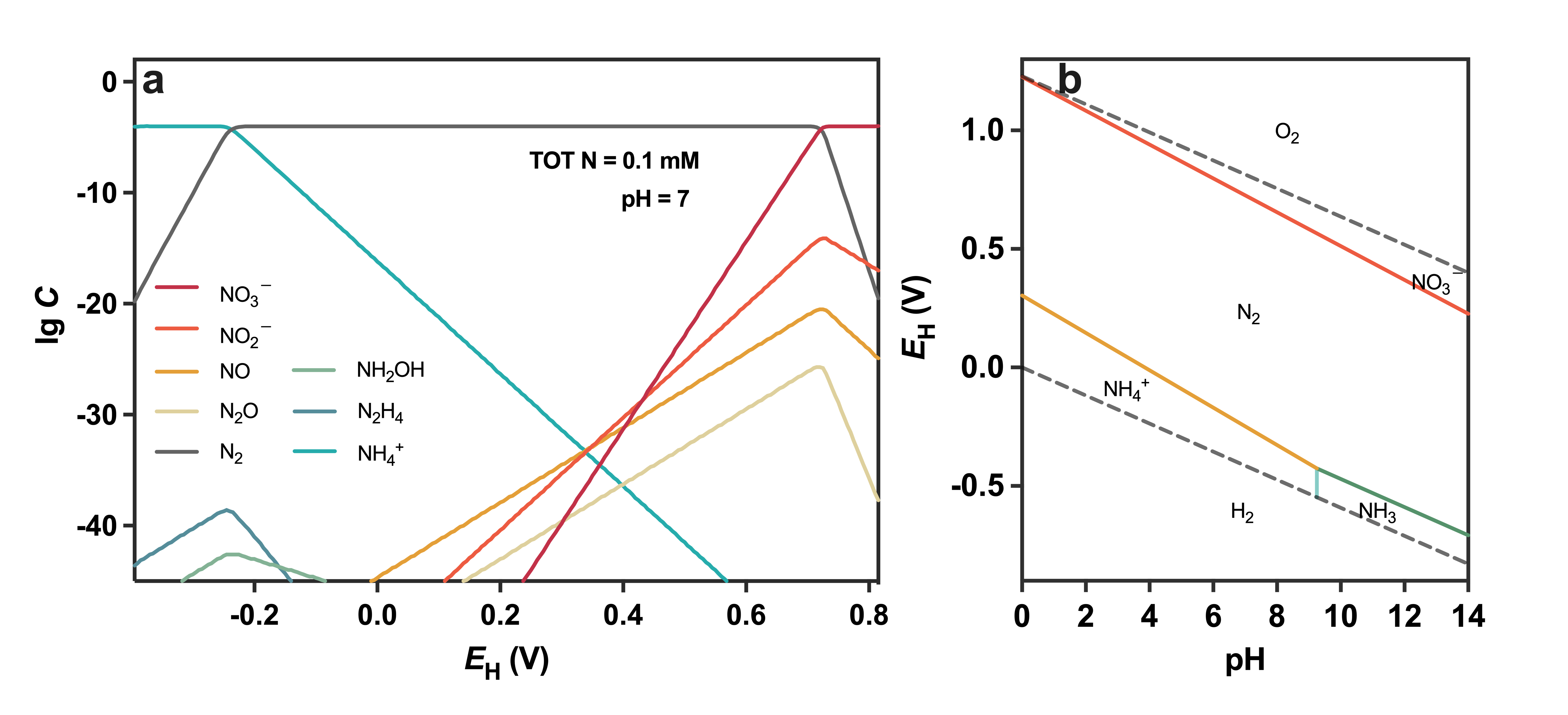


**FIG S1**. (a) lg*C*-*E*_H_ and (b) *E*_H_-pH diagram considering all nitrogen species participating in microbial metabolisms. The total nitrogen concentration (TOT N) was 0.1 mM, which is a typical concentration in natural waters.

**References**

1. van der Star WRL, Dijkema C, de Waard P, Picioreanu C, Strous M, van Loosdrecht MCM. 2010. An intracellular pH gradient in the anammox bacterium Kuenenia stuttgartiensis as evaluated by 31P NMR. Appl Microbiol Biotechnol 86:311–317.

2. Nelson DL, Cox MM, Hoskins AA. 2021. Lehninger Principles of Biochemistry8th ed. W.H. Freeman.

3. Kartal B, Maalcke WJ, de Almeida NM, Cirpus I, Gloerich J, Geerts W, Op den Camp HJM, Harhangi HR, Janssen-Megens EM, Francoijs K-J, Stunnenberg HG, Keltjens JT, Jetten MSM, Strous M. 2011. Molecular mechanism of anaerobic ammonium oxidation. Nature 479:127–130.

4. Brezonik PL, Arnold WA. 2011. Water chemistry: an introduction to the chemistry of natural and engineered aquatic systems. Oxford University Press, New York.

5. Werner S, Morgan JJ. 1996. Aquatic chemistry: chemical equilibria and rates in natural waters, 3rd ed. John Wiley & Sons, Inc., New York.

6. Baalsrud K, Baalsrud KS. 1954. Studies on thiobacillus denitrificans. Arch Mikrobiol 20:34–62.

7. Vogt M. 1965. Wachstumsphysiologische Untersuchungen an Micrococcus denitrificans Beij. Arch Mikrobiol 50:256–281.

8. Straub KL, Benz M, Schink B, Widdel F. 1996. Anaerobic, nitrate-dependent microbial oxidation of ferrous iron. Appl Environ Microbiol 62:1458–1460.

9. Broda E. 1977. Two kinds of lithotrophs missing in nature. Z Für Allg Mikrobiol 17:491–493.

10. Mulder A, Graaf AA, Robertson LA, Kuenen JG. 1995. Anaerobic ammonium oxidation discovered in a denitrifying fluidized bed reactor. FEMS Microbiol Ecol 16:177–184.

11. Strous M, Fuerst JA, Kramer EHM, Logemann S, Muyzer G. 1999. Missing lithotroph identi®ed as new planctomycete. Nature 400:4.

12. Jetten MSM, Wagner M, Fuerst J, van Loosdrecht M, Kuenen G, Strous M. 2001. Microbiology and application of the anaerobic ammonium oxidation (‘anammox’) process. Curr Opin Biotechnol 12:283–288.

13. Oremland RS, Hoeft SE, Santini JM, Bano N, Hollibaugh RA, Hollibaugh JT. 2002. Anaerobic Oxidation of Arsenite in Mono Lake Water and by a Facultative, Arsenite-Oxidizing Chemoautotroph, Strain MLHE-1. Appl Environ Microbiol 68:4795–4802.

14. Rhine ED, Phelps CD, Young LY. 2006. Anaerobic arsenite oxidation by novel denitrifying isolates. Environ Microbiol 8:899–908.

15. Fdz-Polanco F. 2001. New process for simultaneous removal of nitrogen and sulphur under anaerobic conditions. Water Res 35:1111–1114.

16. Cai J, Jiang J, Zheng P. 2010. Isolation and identification of bacteria responsible for simultaneous anaerobic ammonium and sulfate removal. Sci China Chem 53:645–650.

17. Raghoebarsing AA, Pol A, van de Pas-Schoonen KT, Smolders AJP, Ettwig KF, Rijpstra WIC, Schouten S, Damsté JSS, Op den Camp HJM, Jetten MSM, Strous M. 2006. A microbial consortium couples anaerobic methane oxidation to denitrification. Nature 440:918–921.

18. Haroon MF, Hu S, Shi Y, Imelfort M, Keller J, Hugenholtz P, Yuan Z, Tyson GW. 2013. Anaerobic oxidation of methane coupled to nitrate reduction in a novel archaeal lineage. Nature 500:567–570.

19. Costa E, Pérez J, Kreft J-U. 2006. Why is metabolic labour divided in nitrification? Trends Microbiol 14:213–219.

20. van Kessel MAHJ, Speth DR, Albertsen M, Nielsen PH, Op den Camp HJM, Kartal B, Jetten MSM, Lücker S. 2015. Complete nitrification by a single microorganism. Nature 528:555–559.

21. Daims H, Lebedeva EV, Pjevac P, Han P, Herbold C, Albertsen M, Jehmlich N, Palatinszky M, Vierheilig J, Bulaev A, Kirkegaard RH, von Bergen M, Rattei T, Bendinger B, Nielsen PH, Wagner M. 2015. Complete nitrification by Nitrospira bacteria. Nature 528:504–509.

22. Strous M, Kuenen JG, Fuerst JA, Wagner M, Jetten MSM. The anammox case – A new experimental manifesto for microbiological eco-physiology.

23. Clement J, Shrestha J, Ehrenfeld J, Jaffe P. 2005. Ammonium oxidation coupled to dissimilatory reduction of iron under anaerobic conditions in wetland soils. Soil Biol Biochem 37:2323–2328.

24. Huang S, Jaffé PR. 2015. Characterization of incubation experiments and development of an enrichment culture capable of ammonium oxidation under iron-reducing conditions. Biogeosciences 12:769–779.

25. R. C. Aller. 1990. Bioturbation and manganese cycling in hemipelagic sediments. Philos Trans R Soc Lond Ser Math Phys Sci.

26. Su J feng, Zheng SC, Huang T lin, Ma F, Shao SC, Yang SF, Zhang L na. 2015. Characterization of the anaerobic denitrification bacterium *Acinetobacter* sp. SZ28 and its application for groundwater treatment. Bioresour Technol 192:654–659.

27. Wu M, Hou T, Liu Y, Miao L, Ai G, Ma L, Zhu H, Zhu Y, Gao X, Herbold CW, Wagner M, Li D, Liu Z, Liu S. 2021. Novel *Alcaligenes ammonioxydans* sp. nov. from wastewater treatment sludge oxidizes ammonia to N _2_ with a previously unknown pathway. Environ Microbiol 1462-2920.15751.

28. Wu M, Li J, Leu AO, Erler DV, Stark T, Tyson GW, Yuan Z, McIlroy SJ, Guo J. 2022. Anaerobic oxidation of propane coupled to nitrate reduction by a lineage within the class Symbiobacteriia. Nat Commun 13.

29. Garrido-Amador P, Stortenbeker N, Wessels HJCT, Speth DR, Garcia-Heredia I, Kartal B. 2023. Enrichment and characterization of a nitric oxide-reducing microbial community in a continuous bioreactor. Nat Microbiol 1–13.

30. Könneke M, Schubert DM, Brown PC, Hügler M, Standfest S, Schwander T, Schada von Borzyskowski L, Erb TJ, Stahl DA, Berg IA. 2014. Ammonia-oxidizing archaea use the most energy-efficient aerobic pathway for CO _2_ fixation. Proc Natl Acad Sci 111:8239–8244.

31. Bayer B, McBeain K, Carlson CA, Santoro AE. 2023. Carbon content, carbon fixation yield and dissolved organic carbon release from diverse marine nitrifiers. Limnol Oceanogr 68:84–96.

32. Kitzinger K, Koch H, Lücker S, Sedlacek CJ, Herbold C, Schwarz J, Daebeler A, Mueller AJ, Lukumbuzya M, Romano S, Leisch N, Karst SM, Kirkegaard R, Albertsen M, Nielsen PH, Wagner M, Daims H. 2018. Characterization of the First “ *Candidatus* Nitrotoga” Isolate Reveals Metabolic Versatility and Separate Evolution of Widespread Nitrite-Oxidizing Bacteria. mBio 9.

33. Ye J, An N, Chen H, Ying Z, Zhang S, Zhao J. 2020. Performance and mechanism of carbon dioxide fixation by a newly isolated chemoautotrophic strain Paracoccus denitrificans PJ-1. Chemosphere 252:126473.

34. English RS, Williams CA, Lorbach SC, Shively JM. 1992. Two forms of ribulose-1,5-bisphosphate carboxylase/oxygenase from Thiobacillus denitrificans. FEMS Microbiol Lett 94:111–119.

35. Kang D, Li Y, Xu D, Li W, Li W, Ding A, Wang R, Zheng P. 2020. Deciphering correlation between chromaticity and activity of anammox sludge. Water Res 185:116184.

36. Hügler Michael, Wirsen Carl O., Fuchs Georg, Taylor Craig D., Sievert Stefan M. 2005. Evidence for Autotrophic CO2 Fixation via the Reductive Tricarboxylic Acid Cycle by Members of the ε Subdivision of Proteobacteria. J Bacteriol 187:3020–3027.

37. Roden EE, Jin Q. 2011. Thermodynamics of Microbial Growth Coupled to Metabolism of Glucose, Ethanol, Short-Chain Organic Acids, and Hydrogen. Appl Environ Microbiol 77:1907–1909.

38. Strohm TO, Griffin B, Zumft WG, Schink B. 2007. Growth Yields in Bacterial Denitrification and Nitrate Ammonification. Appl Environ Microbiol 73:1420–1424.

39. Vasiliadou IA, Siozios S, Papadas IT, Bourtzis K, Pavlou S, Vayenas DV. 2006. Kinetics of pure cultures of hydrogen-oxidizing denitrifying bacteria and modeling of the interactions among them in mixed cultures. Biotechnol Bioeng 95:513–525.

40. Oshiki M, Satoh H, Okabe S. 2016. Ecology and physiology of anaerobic ammonium oxidizing bacteria. Environ Microbiol 18:2784–2796.

41. Vandekerckhove TGL, Bode S, De Mulder C, Vlaeminck SE, Boon N. 2019. C-13 Incorporation as a Tool to Estimate Biomass Yields in Thermophilic and Mesophilic Nitrifying Communities. Front Microbiol 10.

42. Bayer B, Vojvoda J, Offre P, Alves RJE, Elisabeth NH, Garcia JA, Volland J-M, Srivastava A, Schleper C, Herndl GJ. 2016. Physiological and genomic characterization of two novel marine thaumarchaeal strains indicates niche differentiation. 5. ISME J 10:1051–1063.

43. Li F, Xie W, Yuan Q, Luo H, Li P, Chen T, Zhao X, Wang Z, Ma H. 2018. Genome-scale metabolic model analysis indicates low energy production efficiency in marine ammonia-oxidizing archaea. AMB Express 8.

44. Kits KD, Sedlacek CJ, Lebedeva EV, Han P, Bulaev A, Pjevac P, Daebeler A, Romano S, Albertsen M, Stein LY, Daims H, Wagner M. 2017. Kinetic analysis of a complete nitrifier reveals an oligotrophic lifestyle. Nature 549:269–272.

45. González-Cabaleiro R, Curtis TP, Ofiţeru ID. 2019. Bioenergetics analysis of ammonia-oxidizing bacteria and the estimation of their maximum growth yield. Water Res 154:238–245.

46. Glover HE. 1985. The relationship between inorganic nitrogen oxidation and organic carbon production in batch and chemostat cultures of marine nitrifying bacteria. Arch Microbiol 142:45–50.

47. Hunik JH, Bos CG, van den Hoogen MP, De Gooijer CD, Tramper J. 1994. Co-immobilized Nitrosomonas europaea and Nitrobacter agilis cells: validation of a dynamic model for simultaneous substrate conversion and growth in κ-carrageenan gel beads. Biotechnol Bioeng 43:1153–1163.

48. Ushiki N, Jinno M, Fujitani H, Suenaga T, Terada A, Tsuneda S. 2017. Nitrite oxidation kinetics of two Nitrospira strains: The quest for competition and ecological niche differentiation. J Biosci Bioeng 123:581–589.

49. Hoor AT. 1981. Cell yield and bioenergetics of Thiomicrospira denitrificans compared with Thiobacillus denitrificans. Antonie Van Leeuwenhoek 47:231–243.

50. McComas C, Sublette KL, Jenneman G, Bala G. 2001. Characterization of a Novel Biocatalyst System for Sulfide Oxidation. Biotechnol Prog 17:439–446.

51. Rittmann BE, McCarty PL. 2020. Environmental Biotechnology: Principles and Applications.

52. Strous M, Heijnen JJ, Kuenen JG, Jetten MSM. 1998. The sequencing batch reactor as a powerful tool for the study of slowly growing anaerobic ammonium-oxidizing microorganisms. Appl Microbiol Biotechnol 50:589–596.

53. 2011. Nitrification. ASM Press, Washington, DC.

54. Hayatsu M, Katsuyama C, Tago K. 2021. Overview of recent researches on nitrifying microorganisms in soil. Soil Sci Plant Nutr 67:619–632.

55. Lam P, Kuypers MMM. 2011. Microbial Nitrogen Cycling Processes in Oxygen Minimum Zones. Annu Rev Mar Sci 3:317–345.

56. Upreti K, Rivera-Monroy VH, Maiti K, Giblin AE, Castañeda-Moya E. 2022. Dissimilatory nitrate reduction to ammonium (DNRA) is marginal relative to denitrification in emerging-eroding wetlands in a subtropical oligohaline and eutrophic coastal delta. Sci Total Environ 819:152942.

57. Murphy AE, Anderson IC, Smyth AR, Song B, Luckenbach MW. 2016. Microbial nitrogen processing in hard clam (Mercenaria mercenaria) aquaculture sediments: the relative importance of denitrification and dissimilatory nitrate reduction to ammonium (DNRA). Limnol Oceanogr 61:1589–1604.

58. Chen J-J, Erler DV, Wells NS, Huang J, Welsh DT, Eyre BD. 2021. Denitrification, anammox, and dissimilatory nitrate reduction to ammonium across a mosaic of estuarine benthic habitats. Limnol Oceanogr 66:1281–1297.

59. Borin S, Mapelli F, Rolli E, Song B, Tobias C, Schmid MC, De Lange GJ, Reichart GJ, Schouten S, Jetten M, Daffonchio D. 2013. Anammox bacterial populations in deep marine hypersaline gradient systems. Extremophiles 17:289–299.

60. Byrne N, Strous M, Crépeau V, Kartal B, Birrien J-L, Schmid M, Lesongeur F, Schouten S, Jaeschke A, Jetten M, Prieur D, Godfroy A. 2009. Presence and activity of anaerobic ammonium-oxidizing bacteria at deep-sea hydrothermal vents. ISME J 3:117–123.

61. Zhang Y, Ruan X-H, Op den Camp HJM, Smits TJM, Jetten MSM, Schmid MC. 2007. Diversity and abundance of aerobic and anaerobic ammonium-oxidizing bacteria in freshwater sediments of the Xinyi River (China). Environ Microbiol 9:2375–2382.

62. Jaeschke A, Op den Camp HJM, Harhangi H, Klimiuk A, Hopmans EC, Jetten MSM, Schouten S, Sinninghe Damsté JS. 2009. 16S rRNA gene and lipid biomarker evidence for anaerobic ammonium-oxidizing bacteria (anammox) in California and Nevada hot springs. FEMS Microbiol Ecol 67:343–350.

63. Zhu G, Wang S, Wang Y, Wang C, Risgaard-Petersen N, Jetten MS, Yin C. 2011. Anaerobic ammonia oxidation in a fertilized paddy soil. ISME J 5:1905–1912.

64. Jahangir MMR, Fenton O, Müller C, Harrington R, Johnston P, Richards KG. 2017. In situ denitrification and DNRA rates in groundwater beneath an integrated constructed wetland. Water Res 111:254–264.

65. Ettwig KF, Butler MK, Le Paslier D, Pelletier E, Mangenot S, Kuypers MMM, Schreiber F, Dutilh BE, Zedelius J, de Beer D, Gloerich J, Wessels HJCT, van Alen T, Luesken F, Wu ML, van de Pas-Schoonen KT, Op den Camp HJM, Janssen-Megens EM, Francoijs K-J, Stunnenberg H, Weissenbach J, Jetten MSM, Strous M. 2010. Nitrite-driven anaerobic methane oxidation by oxygenic bacteria. Nature 464:543–548.

66. Jing H, Wang R, Jiang Q, Zhang Y, Peng X. 2020. Anaerobic methane oxidation coupled to denitrification is an important potential methane sink in deep-sea cold seeps. Sci TOTAL Environ 748.

67. Li W, Chen Y, Luo M, Li C, Zhao Y, Chen H, Feng D, Hu B. 2020. Comparison of Anaerobic Methane Oxidation in Different Sediment Habitats of Dianchi Lake. Water Air Soil Pollut 231.

68. Wang Y, Huang P, Ye F, Jiang Y, Song L, Op den Camp HJM, Zhu G, Wu S. 2016. Nitrite-dependent anaerobic methane oxidizing bacteria along the water level fluctuation zone of the Three Gorges Reservoir. Appl Microbiol Biotechnol 100:1977–1986.

69. Shen L, Huang Q, He Z, Lian X, Liu S, He Y, Lou L, Xu X, Zheng P, Hu B. 2015. Vertical distribution of nitrite-dependent anaerobic methane-oxidising bacteria in natural freshwater wetland soils. Appl Microbiol Biotechnol 99:349–357.

70. Zhou L, Wang Y, Long X-E, Guo J, Zhu G. 2014. High abundance and diversity of nitrite-dependent anaerobic methane-oxidizing bacteria in a paddy field profile. FEMS Microbiol Lett 360:33–41.

71. Haaijer SCM, Van der Welle MEW, Schmid MC, Lamers LPM, Jetten MSM, Op den Camp HJM. 2006. Evidence for the involvement of betaproteobacterial Thiobacilli in the nitrate-dependent oxidation of iron sulfide minerals: Thiobacilli and anaerobic oxidation of iron sulfide minerals. FEMS Microbiol Ecol 58:439–448.

72. Böttcher J, Strebel O, Voerkelius S, Schmidt H-L. 1990. Using isotope fractionation of nitrate-nitrogen and nitrate-oxygen for evaluation of microbial denitrification in a sandy aquifer. J Hydrol 114:413–424.

73. Whitmire SL, Hamilton SK. 2005. Rapid Removal of Nitrate and Sulfate in Freshwater Wetland Sediments. J Environ Qual 34:2062–2071.

74. Rios-Del Toro EE, Valenzuela EI, López-Lozano NE, Cortés-Martínez MG, Sánchez-Rodríguez MA, Calvario-Martínez O, Sánchez-Carrillo S, Cervantes FJ. 2018. Anaerobic ammonium oxidation linked to sulfate and ferric iron reduction fuels nitrogen loss in marine sediments. Biodegradation 29:429–442.

75. Kappler A, Schink B, Newman DK. 2005. Fe(III) mineral formation and cell encrustation by the nitrate-dependent Fe(II)-oxidizer strain BoFeN1. Geobiology 3:235–245.

76. Hafenbradl D, Keller M, Dirmeier R, Rachel R, Roßnagel P, Burggraf S, Huber H, Stetter KO. 1996. Ferroglobus placidus gen. nov., sp. nov., a novel hyperthermophilic archaeum that oxidizes Fe2+ at neutral pH under anoxic conditions. Arch Microbiol 166:308–314.

77. Shrestha J, Rich JJ, Ehrenfeld JG, Jaffe PR. 2009. Oxidation of Ammonium to Nitrite Under Iron-Reducing Conditions in Wetland Soils: Laboratory, Field Demonstrations, and Push-Pull Rate Determination. Soil Sci 174:156–164.

78. Yang WH, Weber KA, Silver WL. 2012. Nitrogen loss from soil through anaerobic ammonium oxidation coupled to iron reduction. Nat Geosci 5:538–541.

79. Anschutz P, Sundby B, Lefrançois L, Luther GW, Mucci A. 2000. Interactions between metal oxides and species of nitrogen and iodine in bioturbated marine sediments. Geochim Cosmochim Acta 64:2751–2763.

80. Xu B, Shi L, Zhong H, Wang K. 2021. Investigation of Fe(II) and Mn(II) involved anoxic denitrification in agricultural soils with high manganese and iron contents. J Soils Sediments 21:452–468.

81. Bai Y, Su J, Wen Q, Huang T, Chang Q, Ali A. 2021. Characterization and mechanism of Mn(II)-based mixotrophic denitrifying bacterium (Cupriavidus sp. HY129) in remediation of nitrate (NO3−-N) and manganese (Mn(II)) contaminated groundwater. J Hazard Mater 408:124414.

82. Zhang J, Zhao S, Xu Y, Zhou W, Huang K, Tang Z, Zhao F-J. 2017. Nitrate Stimulates Anaerobic Microbial Arsenite Oxidation in Paddy Soils. Environ Sci Technol 51:4377–4386.

83. Anaerobic Oxidation of Arsenite in Mono Lake Water and by a Facultative, Arsenite-Oxidizing Chemoautotroph, Strain MLHE-1. https://journals.asm.org/doi/epdf/10.1128/AEM.68.10.4795-4802.2002. Retrieved 12 February 2023.
